# Elucidation of a bacterial pathway for catabolism of the β–β-linked dilignol pinoresinol

**DOI:** 10.1128/mbio.02010-25

**Published:** 2025-09-24

**Authors:** Marco N. Allemann, Fachuang Lu, Gerald N. Presley, Hannah R. Valentino, Diana L. Bedgar, Michael A. Costa, Syed G. A. Moinuddin, Christopher C. Azubuike, Delyana P. Vasileva, Dawn M. Klingeman, Leah H. Hochanadel, Alexander R. Fisch, Brian C. Sanders, Lindsay D. Eltis, Richard J. Giannone, Laurence B. Davin, Norman G. Lewis, John Ralph, James G. Elkins, Joshua K. Michener

**Affiliations:** 1 Biosciences Division, Oak Ridge National Laboratory 6146, Oak Ridge, Tennessee, USA; 2 Great Lakes Bioenergy Research Center, Wisconsin Energy Institute, University of Wisconsin 5228, Madison, Wisconsin, USA; 3 Center for Bioenergy Innovation, Oak Ridge National Laboratory 6146, Oak Ridge, Tennessee, USA; 4 Institute of Biological Chemistry, Washington State University 6760, Pullman, Washington, USA; 5 Department of Microbiology and Immunology, The University of British Columbia 8166, Vancouver, British Columbia, Canada; 6 Department of Biochemistry, University of Wisconsin 5228, Madison, Wisconsin, USA; University of Warwick, Coventry, Warwickshire, United Kingdom

**Keywords:** lignin, pinoresinol, *Novosphingobium*

## Abstract

Monolignol-derived dimers containing β–β linkages are synthesized by vascular plants and can be released during lignin depolymerization. In this work, we isolated a bacterium, *Novosphingobium rhizosphaerae* LY, that grows with the β–β lignan (+)-pinoresinol as a sole growth substrate. Sequence analysis suggested that this strain encodes a broad range of pathways for assimilation of aromatic monomers as well as one enzyme implicated in pinoresinol catabolism but lacks other known pathways for aromatic dimer catabolism. We constructed a genome-wide barcoded transposon library and identified genes required for pinoresinol catabolism. Using feeding studies, compound isolation, targeted synthesis, and analysis of purified enzymes, we elucidated the biochemical intermediates and reaction pathway involved in pinoresinol catabolism. We demonstrated that the first enzymatic reaction is the reductive cleavage of a furan ring in (±)-pinoresinol with retention of configuration to yield lariciresinol. We additionally confirmed that the final pathway enzyme, PinU, is related to lignostilbene dioxygenases and oxidatively cleaves a diguaiacylbutadiene intermediate to yield vanillin and coniferaldehyde. Finally, based on the enzyme characterization, we demonstrated that the strain can grow with a second β–β lignan, (–)-syringaresinol, as a sole growth substrate. In combination, these results demonstrate a new biocatalytic route for transforming a widely occurring group of plant phenylpropanoid natural products.

**IMPORTANCE:**

Plants synthesize a variety of aromatic phenylpropanoid compounds containing β–β linkages, including lignin, a major structural polymeric component of the vascular plant cell wall, and lignans, biochemically related secondary metabolites with a wide range of bioactivities. Although microbial catabolic pathways have been described for dimeric phenylpropanoids featuring other interunit linkages, relatively little is known about pathways for catabolism of β–β-linked compounds. In this work, we isolated a *Novosphingobium* strain capable of degrading the β–β-linked lignan (+)-pinoresinol and elucidated the catabolic pathway. Understanding how bacteria catabolize β–β-linked compounds provides a basis for new biocatalytic transformations of lignans and oligolignols and has the potential to improve bacterial lignin valorization.

## INTRODUCTION

Lignin is an aromatic polymer found in vascular plant cell walls and is the most abundant natural aromatic polymer found on Earth (1). The monomeric building blocks of lignin are primarily the hydroxycinnamyl alcohols: *p-*coumaryl, coniferyl, and sinapyl alcohols that differ in the number of methoxy groups present on the aromatic ring (2). These monolignols are polymerized into lignin through radical coupling mechanisms that yield *p*-hydroxyphenyl (H), guaiacyl (G), and syringyl (S) type aromatic units connected by a variety of interunit chemical linkages (3). The proportion of these linkages and the distribution of monomer types in the polymer vary based on the source of the lignin (4).

In attempting to convert biomass into renewable fuels and chemicals, lignin frequently complicates efforts to depolymerize and upgrade cellulosic and hemicellulosic sugars, yet also represents a potential source of value-added products. Lignin can be thermochemically depolymerized into a mixture of aromatic intermediates, either before (5) or after (6) extraction of cellulose and hemicellulose. Interunit linkages with carbon-oxygen bonds, such as the common β–*O*–4 linkage in β-aryl ether units, are relatively easy to break during lignin depolymerization, whereas carbon-carbon linkages are more challenging and are typically retained in dimeric or oligomeric products (7). The resulting complex mixture of aromatic compounds is challenging to process chemically (8) but can be converted by microbes to a smaller range of value-added products, a process described as biological funneling (9–13).

Biological funneling exploits the convergent organization of microbial aromatic catabolic pathways. In the environment, lignin biodegradation is usually initiated by white-rot fungi that partially depolymerize lignin into monomeric and oligomeric products (14). Plants also biosynthesize lower molecular weight polyphenols such as lignans, with these typically being optically active dimers derived from monolignols that often contain interunit linkages similar to those found in lignin (15). Various microbes have evolved a diverse suite of pathways to further catabolize these aromatic compounds as sources of carbon and energy (16, 17). These pathways are typically organized into a set of “upper” pathways that convert a broad range of substrates into a limited number of shared catabolic intermediates. The latter are, in turn, converted to central metabolites by a small number of “lower” pathways. Although individual microbes can catabolize a wide range of aromatic substrates, none is known to assimilate all of the components of depolymerized lignin. To make biological funneling as efficient and economical as possible, microbial cell factories must therefore be engineered with heterologous catabolic pathways to convert additional depolymerization products (18).

Catabolic pathways have not yet been identified for many of the aromatic compounds generated during lignin depolymerization. The best-characterized pathways for catabolism of aromatic compounds are those responsible for monomer catabolism, yet even here, new pathways continue to be discovered (19–24). Even less is known about the catabolism of dilignols. Many of these dimeric compounds, or closely related variants, are produced naturally either as lignans (15), as possible intermediates during lignin polymerization (25), or as can be released as lignin depolymerization products. Given the prevalence of such compounds in the environment, microbes have likely evolved pathways for their catabolism.

**Fig 1:**
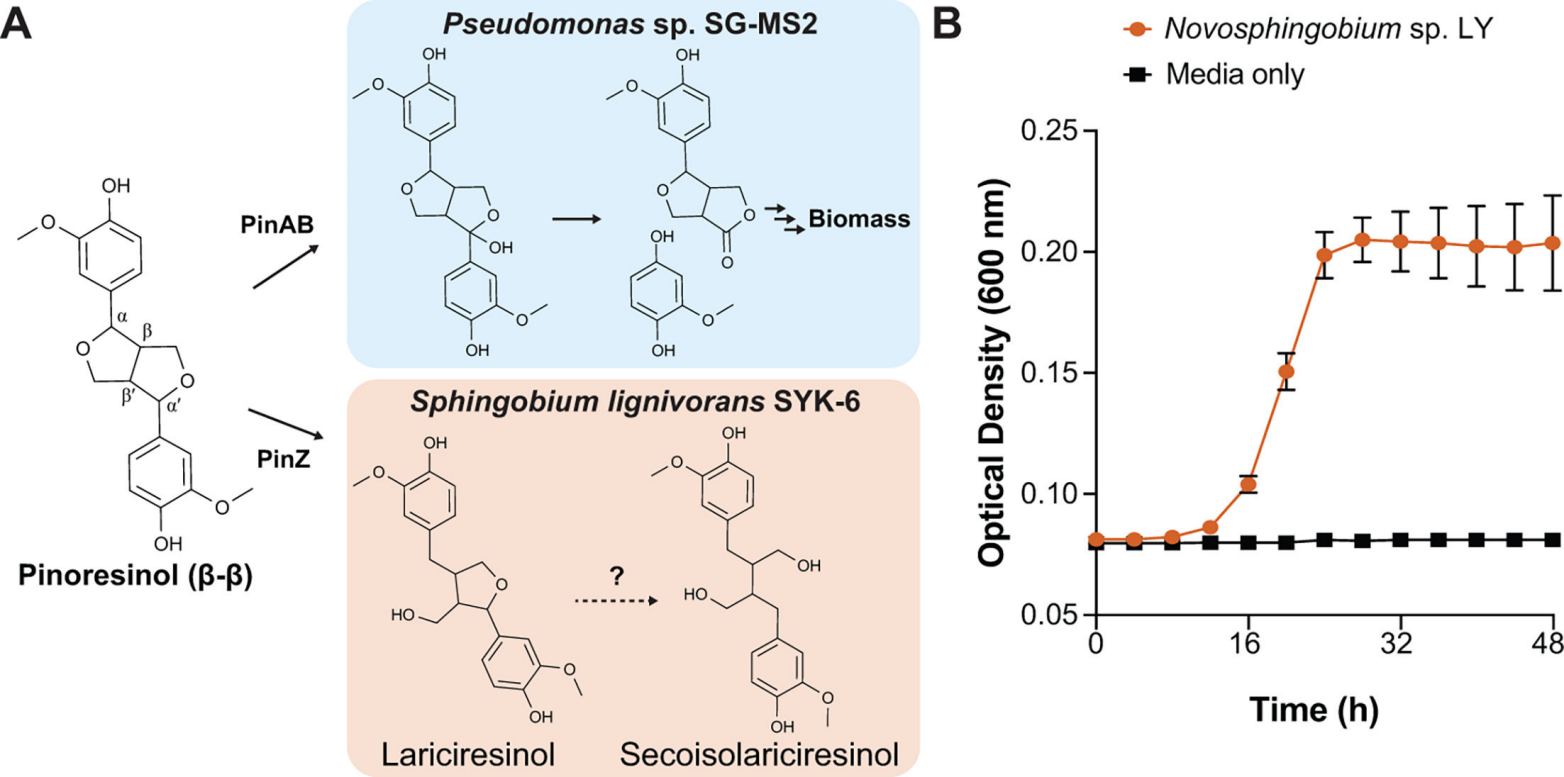
Bacterial pinoresinol catabolism**.** (**A**) Known and putative pathways for pinoresinol catabolism in *Pseudomonas* sp. SG-MS2 and *Sphingobium lignivorans* SYK-6. Reduction of pinoresinol to lariciresinol was observed *in vivo* in SYK-6, whereas further reduction to secoisolariciresinol was only observed *in vitro* using purified PinZ (32). (**B**) The newly isolated strain *N. rhizosphaerae* LY grew with (+)-pinoresinol (red) as the sole substrate, compared to a control without cells (black). Error bars show the standard deviation calculated from three biological replicates.

Pinoresinol is a dilignol that contains two guaiacyl monomers connected by a β–β-type linkage (Fig. 1A). The lignans (+) and (–)-pinoresinol are individually biosynthesized stereoselectively through the action of distinct dirigent proteins (26, 27) and have clinical impacts when consumed (28). Racemic pinoresinol and syringaresinol units are also found in lignin from hardwoods, softwoods, and grasses at typical frequencies of 3%–4% of the total interunit linkages (29–31).

Sphingomonadaceae such as *Sphingobium lignivorans* SYK-6 (hereafter “SYK-6”) and *Novosphingobium aromaticivorans* F199 (also referred to as *N. aromaticivorans* DSM12444, hereafter “F199”) are notable for their ability to catabolize diverse lignin-derived aromatic compounds (33–35). SYK-6 has been used to discover catabolic pathways for model dimeric phenylpropanoids containing β–*O*–4 (guaiacylglycerol–β–guaiacyl ether [GGE]), 5-5 (dehydrodivanillic acid [DDVA]), β–1 (diguaiacylpropanediol [DGPD]), and β–5 (dehydrodiconiferyl alcohol, [DCA]) linkages (36). Similarly, pathways for catabolism of β–*O*–4 and β–1-linked dilignols have been discovered and characterized in F199 (37–39). However, neither strain grows with nor fully degrades pinoresinol (Fig. 1A) (32). Interestingly, one of the two furan rings of pinoresinol is reductively cleaved in SYK-6 by a pinoresinol reductase, PinZ, to form lariciresinol (32). However, no further conversion of lariciresinol was observed *in vivo*.

Recently, strains of *Pseudomonas* sp. SG-MS2 and *Burkholderia* sp. SG-MS1 were isolated based on their ability to grow with (+)-pinoresinol as a sole substrate (40). Although the full details of these pathways have yet to be elucidated, it appears that these strains use an oxidative/hydroxylation mechanism to open the furan ring, with each of the aromatic moieties ultimately being funneled to protocatechuate (PCA) and methoxyhydroquinone (Fig. 1A) (41). Fungi such as *Fusarium solani* also degrade related compounds using similar biochemical transformations (42). Finally, two bacterial strains from the human gut convert pinoresinol to lariciresinol and/or secoisolariciresinol under anoxic conditions, ultimately forming enterodiol or enterolactone (43, 44).

In this work, a novel *Novosphingobium* strain was isolated that grew with (+)-pinoresinol or (–)-syringaresinol as sole substrates. As little was known of the potential catabolic pathway, we used a high-throughput genetic approach based on random barcoded transposon sequencing (RB-TnSeq) to identify potential genes required for β–β dimer catabolism (45, 46), and these were then validated through site-specific deletions. Pathway intermediates were isolated from feeding studies with specific mutants and verified by comparison with standards prepared by chemical synthesis. Selected enzymes were purified and characterized. In combination, these results describe a novel pinoresinol catabolic pathway that can potentially be used for lignin valorization by biological funneling.

## RESULTS AND DISCUSSION

### Isolation and characterization of a pinoresinol-catabolizing sphingomonad strain

We previously isolated bacteria from a variety of environments based on their ability to grow with lignin-related aromatic compounds (manuscript in preparation). The isolates from this culture collection were screened for growth with a commercially available (+)-pinoresinol preparation (88% enantiomeric excess, e.e.), hereafter described as (+)-pinoresinol, as a sole growth substrate. This screen identified one such strain, originally named R3G7B, that, based on its 16*S* rRNA sequence (99.7% nucleotide identity to the type strain) and large yellow colony morphology on nutrient broth plates, we designated as *Novosphingobium rhizosphaerae* sp. LY (hereafter “LY”). This strain grew in solid and liquid 457 minimal medium with (+)-pinoresinol as the sole growth substrate (Fig. 1B). When grown in shake flasks with (+)-pinoresinol, the substrate was fully consumed within 24 hours, consistent with the growth kinetics (Fig. S1A). Further growth assays with variously methoxylated hydroxycinnamates demonstrated that *p*-coumarate, ferulate, sinapate, and syringate all supported growth of LY in minimal medium (Fig. S2). Growth rates and substrate consumption were again consistent during growth with ferulate in shake flasks (Fig. S1B).

### Genome sequence and predicted aromatic catabolic pathways of *N. rhizosphaerae* LY

Genomic DNA was isolated from strain LY and sequenced using a combination of long- and short-read technologies. Genome sequences were annotated by NCBI. The LY genome contains one 4.4 Mbp chromosome and two plasmids of 672 kbp and 149 kbp. The complete genome sequence is deposited in NCBI under accession numbers CP137861–CP137863. Based on sequence annotations and homology searches using known lignin degradation enzymes from SYK-6, LY has a complete protocatechuate 4,5-cleavage pathway along with homologs of several other well-characterized genes for catabolism of aromatic compounds distributed around the genome and plasmids (Table S1). It also has four class III extradiol dioxygenases with unknown substrate specificity that are likely involved in catabolism of additional aromatic compounds (R9J51_04520, R9J51_17410, R9J51_18005, and R9J51_23095). Looking specifically at genes implicated in pinoresinol catabolism, LY has a close ortholog to the SYK-6 *pinZ* gene encoding a pinoresinol reductase (73.3% amino acid identity). LY also contains several orthologs of SYK-6 genes involved in the catabolism of compounds containing β–*O*–4, 5–5, and β–5 linkages, but lacks homologs of genes presumed to be required for complete catabolism of these compounds (Table S1). Consistent with these observations, LY was unable to grow with GGE, which contains a β–*O*–4 linkage, or DDVA, which contains a 5–5 linkage.

### RB-TnSeq and gene deletion identified pinoresinol catabolism genes

**Fig 2:**
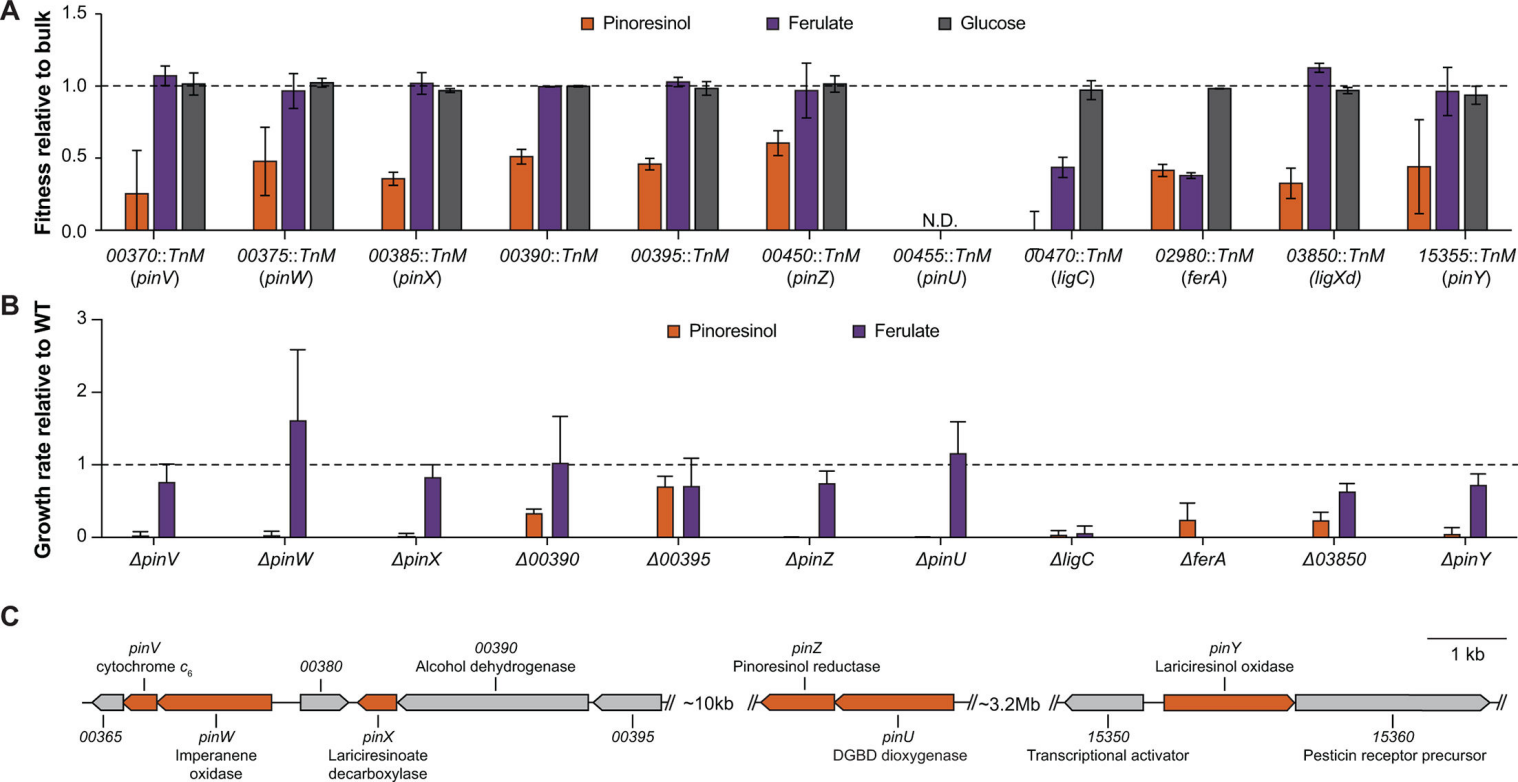
Identification of genes required for (+)-pinoresinol catabolism in *N. rhizosphaerae* sp. LY. (**A**) RB-TnSeq experiments with (+)-pinoresinol, ferulate, and glucose identified genes with pinoresinol-specific fitness defects. Error bars show ±1 standard deviation, calculated from three biological replicates. N.D.: not determined, mutant was not present in the transposon library. Disrupted genes are identified in the figure using their locus tag with the prefix *R9J51*_ deleted for simplicity. (**B**) Clean deletions of putative catabolic genes allowed detailed growth characterization with (+)-pinoresinol and ferulate as growth substrates. Deletions in six genes, annotated as *pinZ* through *pinU*, abolished growth with (+)-pinoresinol but not ferulate. Error bars show one standard deviation, calculated from three biological replicates. (**C**) Genomic context of (+)-pinoresinol catabolic (*pin*) genes (orange) in *N. rhizosphaerae* sp. LY. Functional annotations of non-*pin* genes are based on homology. Unannotated genes are hypothetical.

To identify the genes required for growth of LY with (+)-pinoresinol, we constructed a barcoded *mariner* transposon library, as described previously (37, 46). Based on the sequencing of this library, it consisted of ~64,000 total mutants and contained mapped insertions in 3,463 of 4,708 predicted protein-coding genes. The pooled transposon library was then grown in triplicate in minimal medium with either (+)-pinoresinol, ferulate, or glucose as the sole substrate. We hypothesized that ferulate might be an intermediate in pinoresinol catabolism and, if so, fitness during growth with ferulate could be used to identify genes encoding the lower catabolic pathway. The mixed barcodes were amplified by PCR before and after growth and sequenced to determine the abundances of each barcoded mutant strain under these culture conditions. Fitness values for each gene disruption were calculated as described previously (45, 46), based on the fold change in abundance of barcodes corresponding to transposon insertions in a gene of interest and normalized to the number of generations of each culture (Fig. 2A). As expected, the fitness values during growth with ferulate were low for homologs of genes predicted to be required under those conditions, such as homologs of *ferA* and the enzymes that are predicted to encode the PCA 4,5-cleavage pathway (Fig. S3).

Of the 1,901 genes with differential barcode abundances in this experiment, disruptions in 8 genes led to (+)-pinoresinol-specific fitness effects (Fig. 2A). Consistent with prior characterization in SYK-6, transposon insertions in the *pinZ* homolog, *R9J51_00450*, decreased fitness during growth with (+)-pinoresinol (32). The *pinZ* homolog is also translationally coupled to a gene, *R9J51_00455,* now renamed *pinU*, that is predicted to encode a lignostilbene dioxygenase (Table S1). Lignostilbene dioxygenases are key enzymes in the breakdown of β–5 (47, 48) and β–1 (37, 49, 50) dimers in other sphingomonad strains. This gene was therefore included as a putative target despite not being disrupted in the RB-TnSeq screen. Severe fitness effects were also noted for transposon hits to genes predicted to encode enzymes in the PCA 4,5-cleavage pathway (Table S1 and Fig. S3), which suggested that (+)-pinoresinol is catabolized via PCA. Fitness effects were also noted for transposon hits in the *ferA* homolog (*R9J51_02980*), which suggested that pinoresinol is cleaved to yield a phenylpropanoid intermediate.

To assess the roles of the eight putative pathway genes in the catabolism of pinoresinol, each was deleted in-frame using allelic exchange as described previously (46, 51). The genes *ligC* and *ferA* were also deleted to serve as controls. Individual mutant strains were then assayed for their ability to grow in minimal medium with pinoresinol or ferulate as the sole growth substrate. Six of the eight putative pathway genes were essential for growth in minimal medium containing (+)-pinoresinol as the sole growth substrate but dispensable during growth with ferulate (Fig. 2B). Accordingly, these genes were annotated as *pinZ* through *pinU*. Deletion of two of the genes that displayed fitness effects in the transposon screen during growth with (+)-pinoresinol, *R9J51_00390* and *R9J51_00395*, did not abolish growth with (+)-pinoresinol. Both genes occur upstream of *pinX* in a putative operon (Fig. 2C) and therefore may represent false positives due to polar effects (Fig. 2B). Deletion of a third gene, *R9J51_03850*, reduced but did not abolish growth with (+)-pinoresinol, suggesting that the ferredoxin reductase encoded by this gene participates in but is not essential for pinoresinol catabolism. Consistent with the results from the transposon screen, deletion of the *ligC* homolog was essential for growth with (+)-pinoresinol or ferulate, while deletion of the *ferA* homolog abolished growth with ferulate and decreased growth with (+)-pinoresinol. We did not observe significant fitness effects for disruption of any putative transporters. As pinoresinol is uncharged at relevant pHs, its entry into the cell through passive diffusion may be sufficient to support catabolism (52).

Gene clusters with high sequence identity and synteny to the ~20 kb *pinUZXWV* cluster are present in multiple sequenced isolates of *Novosphingobium capsulatum* and *Novosphingobium pokkalii* that form a monophyletic clade with *N. rhizosphaerae* (53). We observe no association with mobile genetic elements or evidence of horizontal gene transfer for this cluster. Only fragments of this cluster are present in more distantly related sequenced strains. For example, *Novosphingobium* sp. THN1, which shares 97.6% sequence identity in the 16S rRNA gene with *N. pokkalii*, contains homologs of the *pinUZ* and *pinXWV* clusters but separated by more than 1 Mb. Other sequenced isolates contain homologs of only one of the two clusters. The presence of homologs of *pinY* is less consistent, suggesting that this function may be more easily substituted by promiscuous enzyme activities.

### Identification of pathway intermediates

**Fig 3:**
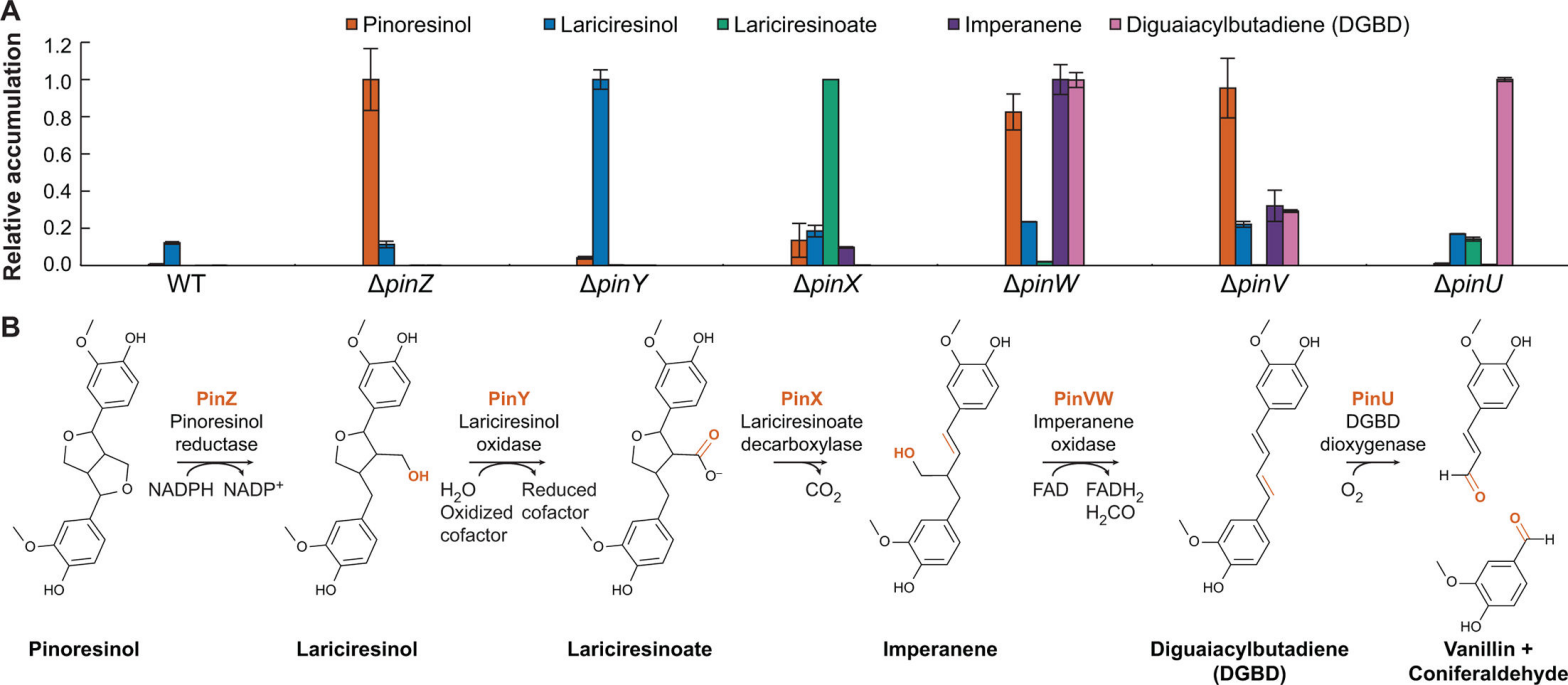
Identification of the pinoresinol degradation pathway. (**A**) Supernatant metabolite concentrations, relative to the maximum observed, after incubating (+)-pinoresinol for 24 h with resting cells of wild-type *N. rhizosphaerae* LY or mutants with clean deletions of the indicated catabolic gene. Substantial DGBD was observed to accumulate in the cell pellet of the ∆*pinU* strain and, therefore, the reported value for this strain is likely an underestimate. Error bars show one standard deviation, calculated from three biological replicates. (**B**) Proposed pinoresinol catabolic pathway in *N. rhizosphaerae* LY. Portions of the metabolites that are modified at each step are highlighted in red.

Although the gene inventory and growth phenotypes provided clues to the associated metabolic pathway, more information was needed to elucidate the sequence of enzymatic transformations. To identify the pathway intermediates, we performed resting cell assays in which each of the *pin* deletion mutants was incubated with (+)-pinoresinol, and the resulting supernatants were analyzed. In preliminary assays, minimal pinoresinol conversion was detected in the ∆*pinZ* mutant, strain JMN132, consistent with PinZ-initiated reduction and previous findings in SYK-6 (32) (Fig. 3A). Next, the *pin* deletion mutants were incubated with (+)-pinoresinol and glucose, and the resulting supernatants were analyzed to detect lariciresinol accumulation. One mutant, JMN131 (∆*pinY*), accumulated higher amounts of lariciresinol compared to the other mutants (Fig. 3A). PinY is a member of the glucose-methanol-choline oxidoreductase family (54) and shares 39% amino acid identity with PhcC from SYK-6, a previously characterized alcohol dehydrogenase involved in the catabolism of dehydrodiconiferyl alcohol (DCA), a model β–5 dimer (55). In the DCA pathway from SYK-6, PhcC performs two successive oxidations of the β-ring side-chain alcohol, yielding a carboxylate. Based on this metabolic logic, we predicted that PinY converts lariciresinol to lariciresinoate (Fig. 3B).

The collection of deletion mutants was then screened for possible accumulation of lariciresinoate using feeding assays with (+)-pinoresinol. Lariciresinoic acid was also synthesized to serve as an analytical standard. One mutant, JMN128 (∆*pinX*), accumulated higher amounts of lariciresinoate relative to other mutant strains (Fig. 3A). Further analysis of the amino acid sequence of PinX indicated that it contains a DUF3237 domain and is 42% and 38% identical at the amino acid level to PhcG and PhcF, two decarboxylases within the phenylcoumaran catabolic pathway from SYK-6 (56). PinX also shares 13% amino acid identity with PadC, a phenolic acid decarboxylase from *Bacillus subtilis* (57). Drawing on the similarities to DCA metabolism in SYK-6, we predicted that PinX catalyzes the decarboxylation of lariciresinoate with concomitant furan ring opening to form imperanene (Fig. 3B).

The prediction of imperanene as a pathway intermediate was confirmed by screening the pathway mutant collection for imperanene-accumulating strains using a synthesized standard. Two mutants, JMN135 (∆*pinV*) and JMN136 (∆*pinW*), accumulated higher amounts of imperanene relative to the other strains (Fig. 3A). PinW contains a predicted FAD-binding domain and shares 34% amino acid identity with the PchF components of the *p*-cresol dehydrogenase flavoprotein subunit from *Pseudomonas putida*, and PinV shares 34% amino acid identity with the cognate PchC cytochrome *c*_6_ (58). Flavoproteins catalyze a variety of reactions and often have a cognate redox partner that acts as an electron donor/acceptor (59). The *pinW* and *pinV* genes are predicted to be co-transcribed and translationally coupled, suggesting that they are involved in the same physiological process. The *p*-cresol dehydrogenase family catalyzes a variety of reactions with lignin-derived aromatic compounds, typically initiated by oxidation to form a quinone methide intermediate (60). For example, the PinAB flavocytochrome from SG-MS2 hydroxylates (+)-pinoresinol through a putative quinone methide intermediate (41). Imperanene accumulation is lower in the ∆*pinV* strain than in the ∆*pinW* strain, suggesting that PinW is required for imperanene conversion, but other proteins may be able to functionally substitute for PinV. Both the ∆*pinV* and ∆*pinW* strains showed low overall conversion of pinoresinol, perhaps due to toxicity or regulatory limitations. We did not attempt to quantify the imperanene concentration in these strains and, therefore, are unable to determine the percent conversion from the fed pinoresinol.

Based on the similarity between the putative intermediate formed by imperanene oxidation and the predicted quinone methide intermediate in β–1 dimer catabolism (38), we hypothesized that a parallel deformylation could yield a diguaiacylbutadiene (DGBD) intermediate (Fig. 3B) via a *p*-quinone methide (Fig. S4). To test this hypothesis, we performed resting cell assays with (+)-pinoresinol and JMN139 (∆*pinU*). The enzyme encoded by *pinU* is homologous to lignostilbene dioxygenases involved in several lignin dimer catabolic pathways (31%–71% amino acid identity, Table S2) (37, 47, 48, 61). These enzymes use molecular oxygen to catalyze the cleavage of an inter-phenyl double bond to yield two aldehydes. The feeding experiments resulted in the accumulation of a pink metabolite that partitioned into the cell pellet fraction. Extraction of the pigmented cell pellet mass yielded a compound that was not present in the wild-type control (Fig. 3A). Using liquid chromatography-mass spectrometry (LC-MS), this compound was identified as DGBD by comparison to a synthesized standard (Fig. S5). Quantification of DGBD was inexact, as the compound largely coprecipitated with the cell pellet. Despite yielding comparable measured DGBD concentrations (Fig. 3A), the cell pellets of the ∆*pinV* and ∆*pinW* strains were not visibly pink, suggesting that DGBD accumulation in these strains was significantly lower than in the ∆*pinU* strain. Due to the symmetry of DGBD, cleavage of either of the benzylic double bonds by PinU is predicted to yield vanillin and coniferaldehyde (Fig. 3B). As deletion of *R9J51_00390* decreased but did not abolish growth with pinoresinol (Fig. 2B), we hypothesized that it might affect assimilation of vanillin or coniferaldehyde. However, there were no significant differences in metabolite accumulation in JMN137 (∆*R9J51_00390*) and wild-type LY, suggesting that the protein encoded by *R9J51_00390* has another role.

### PinZ reduces each pinoresinol enantiomer with retention of configuration

To further validate the proposed pinoresinol catabolic pathway and to assess its potential substrate range, we expressed and purified the first enzyme in the pathway, PinZ, from *E. coli* (Fig. S6). We incubated recombinant PinZ with either racemic (±)-pinoresinol or a preparation enriched in (+)-pinoresinol (~88% e.e.) in the presence of NADPH and analyzed the resulting conversions using chiral-phase HPLC. Authentic synthetic standards of (±)-pinoresinols and (±)-lariciresinols produced well-separated enantiomers by this analysis, whereas the (±)-secoisolariciresinols were not resolved under these conditions (Fig. S7).

**Fig 4:**
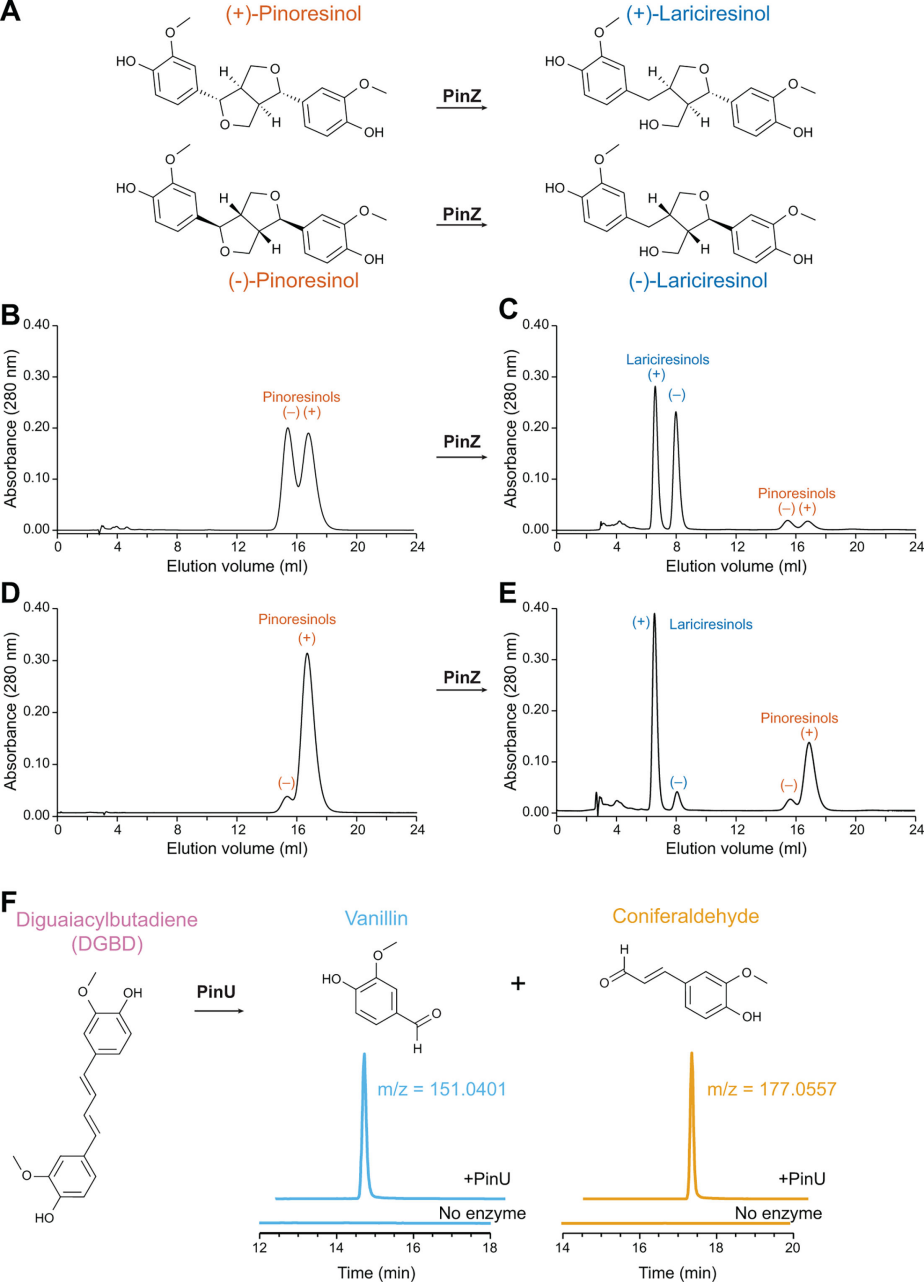
Determination of the reaction products of PinZ- and PinU-catalyzed reactions**.** (**A**) The pinoresinol reductase PinZ converts pinoresinol to lariciresinol. To assess the enantioselectivity and enantiospecificity of PinZ, either (**B**) racemic pinoresinol or (**D**) a preparation enriched in (+)-pinoresinol (~88% e.e.) was incubated with recombinant PinZ and 10 mM NADPH for 10 minutes at 30°C and then analyzed by chiral HPLC. The two enantiomers were reduced at equivalent rates (**C and E**) with retention of configuration. (**F**) Purified PinU was incubated with isolated DGBD and conversion was monitored by HPLC-MS at the indicated *m*/*z* ratios. Monomer formation was only observed in the presence of PinU.

Racemic pinoresinol was reduced to racemic (±)-lariciresinol, and the ~88% e.e (+)-pinoresinol was reduced to (+)-lariciresinol (~84% e.e) (Fig. 4B through E; Table S3), indicating that both enantiomers of pinoresinol were reduced with retention of configuration. However, neither (+)- nor (–)-secoisolariciresinols were formed. This ability of PinZ to act with both (+)- and (–)-pinoresinol enantiomers differs from the well-characterized pinoresinol and pinoresinol-lariciresinol reductases (PRs and PLRs) in vascular plant species (62–65). PinZ’s reductive capabilities may be relevant to pinoresinol catabolism of both lignans and lignins.

Apparent steady-state kinetic parameters of PinZ were next determined using racemic (±)-pinoresinol and the ~88% e.e. (+)-pinoresinol (Fig. S8 and Table S4) in the presence of NADPH; NADH did not serve as a cofactor (data not shown). The parameters for the two substrates were remarkably similar, with apparent *K_m_* values of 27.0 ± 6.6 µM and 22.1 ± 3.2 µM, respectively, and apparent specificity constants *k*_cat_*/K_m_* of 4.69 × 10^5^ and 4.86 × 10^5^ M^−1^ s^−1^. That is, the enzyme had no discernible preference for either of the two enantiomers. Compared to recombinant plant PLRs/PrRs, PinZ had a higher *k*_cat_, whereas the *K_m_* was similar or higher (62, 65, 66) (Table S4). These functional differences are consistent with differing roles in catabolism and biosynthesis.

The total activity of recombinant PinZ, expressed as pkat/µg protein, was similar to that reported for PinZ from SYK-6 (32). These similarities suggest that SYK-6 was unable to grow with pinoresinol because it lacks the enzymes for lariciresinol catabolism rather than having a deficiency in PinZ activity.

Characterization of PinZ with additional resinol substrates, reported separately, demonstrated that PinZ could reduce racemic (±)-medioresinol and racemic (±)-syringaresinol with similar or higher catalytic efficiencies as compared to racemic (±)-pinoresinol (Smith et al., unpublished data). Therefore, we also measured the growth of LY with (–)-syringaresinol as a sole growth substrate. Wild-type LY grew with (–)-syringaresinol, though with a longer lag phase and lower final density compared to growth with (+)-pinoresinol (Fig. S9). Deletion of *pinZ* abolished growth with both (–)-syringaresinol and (+)-pinoresinol (Fig. S9), suggesting that the initial step of catabolism is the same for both substrates.

### PinU oxidatively cleaves DGBD to yield monomeric products

To validate the reaction catalyzed by PinU and confirm the products of pinoresinol catabolism in LY, we produced PinU in *E. coli* and partially purified it (Fig. S10). DGBD was isolated through ethyl acetate extraction of JMN139 (LY ∆*pinU*) grown in the presence of pinoresinol, purified by flash chromatography, and its structure verified by NMR spectroscopy (Fig. S11 and 12). We then incubated purified DGBD with PinU and monitored product formation by LC-MS/MS analysis. Incubation with PinU led to the formation of vanillin and coniferaldehyde, consistent with oxidative cleavage of one of the two benzylic double bonds (Fig. 4F; Fig. S13 and 14). Pathways for catabolism of these monomers have been described previously, for example, by oxidation of coniferaldehyde to ferulate (67, 68).

### Conclusion

Bioprospecting efforts in combination with strain characterization and subsequent high-throughput genetic screenings allowed us to rapidly identify and characterize a complete β–β lignin catabolism pathway in *N. rhizosphaerae* sp. LY. This pathway is unrelated to the previously characterized pathway found in *Pseudomonas* sp. SG-MS2 and *Burkholderia* sp. SG-MS1. Initial studies with purified enzymes revealed that the first reaction, catalyzed by PinZ, is not enantiospecific. Analysis of the final enzyme, PinU, confirmed that (+)-pinoresinol is ultimately converted to monomeric intermediates vanillin and coniferaldehyde. Although further detailed enzymology studies will be required to clarify specific details of each biochemical transformation in this pathway, the discovery of the required genes will allow this pathway to be expressed in new hosts to support future microbial lignin valorization efforts.

## MATERIALS AND METHODS

### Bacterial strains and growth conditions

*N. rhizosphaerae* sp. LY strains were routinely grown at 30°C in 25% strength (2 g/L) nutrient broth (NB) medium (Difco). Minimal defined medium for *N. rhizosphaerae* sp. LY was medium 457 (DSMZ) with 0.2 g/L glucose or 0.2% (wt/vol) casamino acids as a sole growth substrate. Most aromatic carbon substrates were dissolved in dimethylsulfoxide (DMSO) and added to minimal medium at 1 g/L final concentration. Due to the insolubility of pinoresinol in minimal medium, it was added to a final concentration of 0.1 g/L.

Unless otherwise specified, *Escherichia coli* strains were grown in lysogeny broth at 37°C. For solid media, agar was included at 15 g/L. When necessary, diaminopimelic acid (DAP) was used at 60 µg/mL. For conjugation experiments using both *N. rhizosphaerae* and *E. coli,* the antibiotics kanamycin (50 µg/mL) and streptomycin (100 µg/mL) were used as required. Strains and plasmids used in this work are described in Table S5.

### Lignans and related standards

Enantiomerically enriched (+)-pinoresinol (~88% enantiomeric excess, hereafter called (+)-pinoresinol), (+)-lariciresinol, and (–)-syringaresinol preparations were purchased from Cfm Oskar Tropitzsch (Marktredwitz, Germany), Biosynth (Staad, Switzerland), and AvaChem (San Antonio, Texas), respectively. Guaiacylglycerol-β-guaiacyl ether (GGE) was purchased from TCI Chemicals (Tokyo, Japan). Syntheses of lariciresinoic acid (Fig. S15 and 16), imperanene, and diguaiacylbutadiene are reported in the Supplementary Methods. Racemic (±)-pinoresinol, (±)-lariciresinol, and (±)-secoisolariciresinol were synthesized as reported (69).

### Isolation of *Novosphingobium* strains

Sediment was isolated from a natural hot spring near Rabbit Creek in Yellowstone National Park on June 18th, 2018, at coordinates 44.51347, −110.8148. The spring temperature near the sediment isolation was measured at 57.4°C and the pH of the water in the sediment sample was 7.5. Sediment samples were used to inoculate sterile M9 minimal medium (6 g/L Na_2_HPO_4_, 3 g/L KH_2_PO_4_, 0.5 g/L NaCl, 1 g/L NH_4_Cl, 1 mM MgSO_4_, 0.1 mM CaCl_2_; Sigma-Aldrich) containing 1 g/L GGE. A sediment slurry was diluted 100-fold into fresh medium, and the cultures were incubated at 30°C with shaking at 200 rpm until they grew to saturation. Six successive passages into fresh medium were performed prior to plating a dilution of the isolates onto M9 minimal medium agar containing 1 g/L GGE. A single colony was picked from plates and streaked onto R2A agar for further isolation. An isolated colony (R3G7B) was inoculated into R2A broth and grown overnight at 30°C prior to freezing into cell stocks. Culture stocks were preserved in 25% (vol/vol) glycerol at –80°C. Despite being isolated with GGE as the sole growth substrate, R3G7B was unable to grow with GGE as a pure culture. This discrepancy may be the result of cross-feeding during isolation or reflect abiotic degradation of the substrate used during the enrichments.

### Genome sequencing

Genomic DNA was obtained from wild-type *N. rhizosphaerae* sp. LY using a phenol-chloroform-based extraction method (70) then concentrated by ethanol precipitation (71). PacBio sequencing was performed by SNPsaurus (Eugene, OR). For strain resequencing to confirm genetic variations, Nextera XT libraries (Illumina, San Diego, CA) were made according to the manufacturer’s instructions (15031942 v05). Libraries were validated using a DNA 7500 chip on an Agilent Bioanalyzer (Agilent, Santa Clara, CA). Barcoded libraries were pooled based on concentrations obtained by Qubit (Invitrogen, Waltham, MA). Paired-end sequencing (2 × 301) was performed on an Illumina MiSeq (Illumina, San Diego, CA) using v3 chemistry.

### Transposon mutagenesis of *N. rhizosphaerae* sp. LY

A barcoded *mariner* transposon library (pKMW3) was mobilized into *N. rhizosphaerae* sp. LY via biparental conjugation using *E. coli* WM3064 as described previously (45, 46). An aliquot of the WM3064 pKMW3 library was thawed on ice and inoculated into LB supplemented with DAP and kanamycin and grown to late log phase at 37°C. The cells were harvested by centrifugation and washed three times with 25% strength NB. Washed *E. coli* donor cells and *N. rhizosphaerae* sp. LY cells from an overnight culture were mixed in a 1:5 ratio (vol/vol), respectively, and collected by centrifugation. The cell mixture was resuspended in a minimal volume of medium and spotted onto 25% strength NB agar supplemented with DAP. Once the cell mixture dried, the plates were incubated at 30°C overnight. Cell spots were scraped off plates and resuspended in phosphate-buffered saline (PBS) and rinsed three times with PBS before being plated on 25% strength NB agar plates supplemented with kanamycin to select for transposon mutants. After 5 days incubation at 30°C, colonies were scraped off plates, and the resulting pool was diluted 100-fold into NB supplemented with 50 µg/mL kanamycin and grown overnight at 30°C. Aliquots of the culture were frozen at −80°C by the addition of glycerol (25% [wt/vol] final).

Genomic DNA from the pooled transposon library was isolated and used for transposon mapping as described previously (45, 46). DNA was sheared by sonication (Bioruptor Plus Diagenode, Ougrée, Belgium) and double size selected to approximately 300 bp fragments with AMPure beads (Beckman Coulter, Brea CA) following the manufacturer’s instructions. Size-selected DNA was end-repaired (NEB E6050), dA-tailed (NEB6053), and adaptors were ligated (NEB E6056), following the manufacturer’s instructions. DNA was bead-purified with AMPure beads between each modification. For adaptor ligation, 0.5 µL of a 15 µM double-stranded Y adapter was used (45). Transposon enrichment was completed with 25 cycles of polymerase chain reaction (PCR) using KAPA HiFi Hotstart ready mix (Roche, Indianapolis IN). The enrichment reaction was purified with 0.8× AMPure beads. The final library was assessed for quality on an Agilent Bioanalyzer (Santa Clara, CA) and a Thermo Fisher Nanodrop instrument (Waltham, MA). Concentration was determined on an Invitrogen Qubit (Waltham, MA) with the broad range double-stranded DNA assay. Final libraries were denatured and diluted following the Illumina guidelines, and sequencing was completed on an Illumina MiSeq instrument (Illumina, San Diego, CA). Read lengths were 151 bp with V2 chemistry. Fitness values for gene disruptions were calculated as described previously (45), roughly corresponding to the average log_2_ fold change in abundance of barcodes representing the mutants with transposon insertions in the middle 80% of the gene. These values were then normalized to the number of doublings of the bulk population, as described previously (46). Gene homologs were identified using BLAST (72) and aligned using Geneious Prime 2025.1.2.

### Allele exchange mutagenesis in *N. rhizosphaerae* sp. LY

Mutants of *N. rhizosphaerae* sp. LY were generated by allele exchange using the plasmid pAK405 as described previously (51). For in-frame deletions, homologous regions (~700 bp) upstream and downstream of the target gene were synthesized and cloned onto pAK405 (Genscript, New Jersey). Deletion constructs were designed to generate in-frame deletions and maintain the start and stop codons of the target gene to minimize possible polar effects. Constructs were mobilized into *N. rhizosphaerae* sp. LY via biparental conjugation using the *E. coli* DAP auxotroph strain WM6026 with selection on quarter-strength NB medium containing kanamycin (50 µg/mL). Exconjugants were streaked to single colonies once from primary selection plates and grown overnight in liquid NB in the absence of kanamycin selection. Aliquots of the overnight culture were plated on NB + streptomycin agar for counterselection against the integrated plasmid. Streptomycin-resistant colonies were patched to NB + kanamycin + streptomycin and NB + streptomycin to screen for kanamycin sensitivity. Kanamycin-sensitive colonies were screened for gene deletions by colony PCR and then verified by whole-genome resequencing.

### Growth rate measurements

Strains were grown to saturation (OD_600_ ~0.4–0.5) in DSM 457 minimal medium with 2 g/L glucose at 30°C. Cells were pelleted and washed with DSM 457 lacking a growth substrate before being diluted 100-fold into fresh medium containing the specified substrate and grown as triplicate 1 mL cultures in 48-well plates (Greiner). Growth at 30°C was monitored by measuring the optical density at 600 nm in a BioTek Epoch2 plate reader with readings taken every 15 min for a total of 120 h. The growth rates were calculated using the R package growthcurver, which calculates growth rates based on a logistic growth model using the entire data set as input (73).

### Resting cell assays

The appropriate wild-type or mutant derivatives of *N. rhizosphaerae* sp. LY were grown overnight in DSM 457 mineral medium supplemented with 0.2% (wt/vol) casamino acids as a growth substrate at 30°C. Aliquots of the cultures were pelleted by centrifugation, and the cells were washed several times with DSM 457 medium lacking any growth substrate. Washed cells were resuspended to an OD_600_ of approximately 0.4 in DSM 457 supplemented with 0.1 g/L (+)-pinoresinol and incubated at 30°C for 24 h. After incubation, 1 mL aliquots were removed, and cells were pelleted by centrifugation. The cell-free culture supernatant was then passed through a 0.2 µm filter and stored at −80°C for later analysis.

### Diguaiacylbutadiene extraction from *N. rhizosphaerae* LY cell pellets

Pellets of JMN139 (LY ∆*pinU*) incubated with (+)-pinoresinol were resuspended in 10 mL ethyl acetate and lysed on ice for 2 min by ultrasonication with pulse intervals of 10 s at 50% amplitude using a Branson SFX 250 Sonifier. The ethyl acetate mixture was then centrifuged for 10 min at 1,000 × *g*. The ethyl acetate supernatant was pipetted from the pelleted cell debris, and the ethyl acetate was removed under reduced pressure to obtain the dried extract. The extract was purified using a Biotage Selekt high-performance automated flash chromatography system with a Biotage Sfär Silica D Duo 60 µm 5 g column. The crude dried extract was then dissolved in acetone, and 2.0 g of silica-gel chromatography powder was added to form a slurry mixture with the extract. The acetone was removed using a rotary evaporator under reduced pressure, and the solid silica-loaded extract was placed on the column. A linear gradient mobile phase was applied to the column, starting with 100% dichloromethane and ending in 100% methanol. Total run time was 24 min with a flow rate of 80 mL/min. Diguaiacylbutadiene was collected across two 20 mL fractions, and the solvent was removed by rotary evaporator under reduced pressure to afford 3.0 mg of a pink solid that was characterized via ^1^H NMR spectrometry on the Avance III 400 MHz NMR Spectrometer (Fig. S9 and 10). The solid material was dissolved in CDCl_3_ for the analysis.

### HPLC-MS analyses

Culture supernatant from wild-type strains and mutant derivatives of *N. rhizosphaerae* sp. LY incubated with (+)-pinoresinol was analyzed by LC-MS/MS to confirm and quantify pathway bottlenecks resulting from targeted gene deletions. As described above, standards were either purchased or synthesized and measured by targeted parallel reaction monitoring (PRM) to assess specific analyte fragmentation profiles. LC-MS/MS measurements were performed on a Q Exactive Plus mass spectrometer (Thermo Fisher Scientific) interfaced directly to a Vanquish uHPLC (Thermo Fisher Scientific). For each knockout strain, 5 µL of supernatant was split-loaded onto an in-house pulled nanospray emitter (75 µm inner diameter) packed with 15 cm of C18 resin (1.7 µm Kinetex; Phenomenex) and separated over a 15 min reversed-phase gradient as previously described (37). Eluting analytes were measured by the Q Exactive operating in negative-ion mode with a duty cycle that included a full scan (100–550 *m*/*z* range; resolution 35,000; 3 microscan spectrum averaging) followed by data-dependent fragmentation (MS/MS) of supernatant analytes (resolution 17,500; higher-energy collisional dissociation fragmentation at 30/35/40% averaged normalized collision energy). Similar conditions were employed for targeted PRM measurements of the analytical standards. Peak areas were extracted for all pathway analytes/intermediates via Skyline software (74) and areas compared across samples.

### PinZ expression and purification

The full-length *pinZ* coding sequence from *N. rhizosphaerae* LY was synthesized and cloned (Genscript) into the pET-28a(+) backbone (Novagen) between the *Nde*I and *Xho*I restriction sites to yield plasmid pJM492 that expresses PinZ with an N-terminal hexahistidine tag. This plasmid was transformed into One Shot BL21 Star (DE3) competent *E. coli* (Invitrogen) according to the manufacturer’s protocol. An initial lysogeny broth (LB) medium culture (10 mL) containing 25 µg/mL kanamycin was incubated overnight (approximately 15 h) at 37°C with shaking at 250 rpm. A 500 µL aliquot of each culture was then used to inoculate LB medium (50 mL) containing 25 µg/mL kanamycin. After incubating at 37°C with shaking at 250 rpm to obtain an OD_600_ of approximately 0.6, the cultures were induced with isopropyl β-D-1-thiogalactopyranoside (IPTG) at a final concentration of 0.25 mM. After continual shaking at 15°C for 24 h, the culture medium was divided between five centrifuge tubes, with cells harvested by centrifugation at 3,000 × *g* for 20 min at 4°C, with the pellet frozen and stored at –80°C.

The pelleted culture (one tube from above) was defrosted, then lysed using BugBuster Protein Extraction Reagent (EMD Millipore, 1 mL) containing Benzonase Nuclease (EMD Millipore, 1 µL) and rLysozyme (EMD Millipore, 0.2 µL). After shaking (rocking shaker, 10 min), the suspension was centrifuged (16,000 × *g*, 10 min), and the supernatant was removed. Purification used a POROS 20 MC metal chelate affinity column (~500 µL bed volume) equilibrated in binding buffer (20 mM Tris-HCl, pH 7.9, 500 mM NaCl). After loading the crude protein extract, the column was washed with six bed volumes of binding buffer to remove unbound proteins, followed by four bed volumes of binding buffer containing 15 mM imidazole. Hexahistidine-tagged (ht-) PinZ was next eluted with eight bed volumes of binding buffer containing 100 mM imidazole.

Individual fractions were subjected to SDS-PAGE using a Mini-PROTEAN TGX Precast Gel, 4–20% gradient (Bio-Rad), with visualization by silver staining (Fig. S6). Fractions containing PinZ were pooled, concentrated (Amicon Ultra-2 10K centrifugal filter [Millipore]), and exchanged into 20 mM Tris-HCl, pH 7.0. After concentration, protein was quantified using the Bradford micro-assay (Bio-Rad) procedure.

### PinZ enzyme assays

PinZ assays were performed with racemic pinoresinol or a (+)-pinoresinol preparation. Assays were individually performed in 250 µL, consisting of buffer (20 mM Tris-HCl, pH 7.0, 185 µL), substrates (5 mM, 20 µL), NADPH (10 mM, 40 µL), and PinZ (5 µL, 18 ng). Reactions were incubated at 30°C for 10 min, stopped by the addition of glacial acetic acid (10 µL), extracted with ethyl acetate (2 × 0.5 mL), and dried *in vacuo*. For reversed-phase chromatography, dried assay extracts were resuspended in 50 µL methanol. A 10 µL aliquot of each reaction was injected. For corresponding chiral separations, dried extracts were resuspended in 100 µL ethanol, and 80 µL aliquots were injected. Assays were also carried out in the presence of NADH (10 mM, 20 µL) as described above for NADPH; however, no enzymatic activity was observed.

HPLC separations were performed on an Alliance 2690 HPLC system (Waters, Milford, MA) equipped with UV–Vis diode-array detector (Model 2990, Waters), with detection at 280 nm. Reversed-phase separations used a Symmetry Shield RP_18_ column (150 × 3.5 mm, Waters) eluted with A (3% acetic acid in H_2_O) and B (acetonitrile) at a flow rate of 1 mL/min as follows: 95:0 from 0 to 2 min followed by linear gradients from 95:0 to 85:15 in 3 min, 85:15 to 60:40 in 25 min, 60:40 to 5:95 in 5 min after which the column was re-equilibrated to the starting conditions. Chiral-phase chromatographic separations of (±)-pinoresinols and (±)-lariciresinols were carried out on a Chiralcel OC column (250 × 4.6 mm, Chiral Technologies, West Chester, PA) eluted with EtOH-hexanes (8:2, v/v) at a flow rate of 0.2 mL/min.

For steady-state kinetic analyses, assays were performed as described above using 10 different substrate concentrations (0.5–400 µM). Reactions were performed in triplicate and analyzed by reversed-phase HPLC. The Hyperbola function in Origin was used to determine the Michaelis-Menten kinetics parameters (*K*_*m*_ and *V*_max_) (Fig. S8).

### PinU expression and purification

The full-length *pinU* open-reading frame from *N. rhizosphaerae* sp. LY was synthesized and cloned (Genscript) into the pET-24a(+) backbone (Novagen) between the *Nde*I and *Xho*I restriction sites to yield plasmid pJM491. PinU was produced heterologously in *E. coli* BL21(DE3) harboring the expression plasmid. Transformed cells were grown at 37°C in LB supplemented with 50 µg/mL kanamycin to a turbidity (at 600 nm) of 0.6–0.8, and then expression of the protein was induced by the addition of 1 mM IPTG, followed by further overnight incubation at 30°C. At the time of induction, the medium was supplemented with 0.5 mM FeCl_3_ as described previously (48).

PinU was partially purified at 4°C. Cells were harvested by centrifugation and suspended in 20 mM 4-(2-hydroxyethyl)-1-piperazinepropanesulfonic acid (HEPPS), pH 8.0. A crude extract was then prepared by sonication and centrifugation. The supernatant was applied to a HiTrap Q FF anion exchange chromatography column (GE Healthcare) using an ÄKTA FPLC instrument (GE Healthcare), following the manufacturer’s recommendations. The protein was eluted with a linear NaCl gradient (0.3–1 M) in 20 mM HEPPS buffer, pH 8.0, and collected in 5 mL fractions. The fractions were analyzed for purity using NuPAGE 4–12% Bis-Tris protein gels and the corresponding NuPAGE MOPS SDS running buffer (Thermo Scientific) with visualization by Coomassie Blue (Fig. S10). Fractions containing PinU were pooled and exchanged into 20 mM HEPPS, pH 8.0, using an Amicon Ultra 30K device (Merck Millipore). Protein concentrations were estimated using Bradford Protein Assay Kit (OZ Biosciences) using bovine serum albumin as a standard. Fresh samples of PinU were used in all enzyme assays.

### PinU enzyme assays

PinU assays were performed in 1 mL of 40 mM 4-(2-hydroxyethyl)piperazine-1-ethane-sulfonic acid (HEPES), pH 7.5. Stock solutions of the substrate were prepared in DMSO, and reactions were initiated by the addition of purified DGBD. Samples were analyzed using PRM LC-MS/MS, specifically targeting vanillin ([M–H]^–^ at 151.0401 *m/z*) and coniferaldehyde ([M–H]^–^ at 177.0557 *m/z*), using the same instrumentation and HPLC conditions as described above. Target ions were isolated within a 1.0 *m/z* window, automatic gain control of 2 × 10^5^ ions with 100 ms max fill time, 17,500 resolution, and higher energy collisional dissociation fragmentation at 30/35/40% averaged normalized collision energy. Fragmentation profiles were matched to previously measured standards as well as MS/MS deposited in the MassBank of North America (MoNA; https://mona.fiehnlab.ucdavis.edu/). Peak areas were extracted for both analytes via Skyline software (74) and areas compared across samples to confirm the presence of vanillin and coniferaldehyde with PinU addition.

## Data Availability

The genome sequence of *N. rhizosphaerae* is available under GenBank accessions CP137861–CP137863. Raw sequencing data are available from BioProject PRJNA1034628.

## SUPPLEMENTAL MATERIAL

Supplemental Information mbio.02010-25-s0001.pdf Figures S1-S16; Tables S1-S5.

ASM does not own the copyrights to Supplemental Material that may be linked to, or accessed through, an article. The authors have granted ASM a non-exclusive, world-wide license to publish the Supplemental Material files. Please contact the corresponding author directly for reuse.
