# Supplementary material for: Elucidation of a bacterial pathway for catabolism of the β–β-linked dilignol pinoresinol
Source: mBio. 2025 Sep 24;16(11):e02010-25. doi: 10.1128/mbio.02010-25 (PMC12607616; doi:10.1128/mbio.02010-25)
Supplement: Supplemental Information — Figures S1-S16; Tables S1-S5. [file mbio.02010-25-s0001.pdf]

Supplemental Information

Elucidation of a bacterial pathway for catabolism of the β–β-linked dilignol pinoresinol

Marco N. Allemann[1], Fachuang Lu[2], Gerald N. Presley[1,3,‡], Hannah R. Valentino[1], Diana L. Bedgar[4], Michael A. Costa[4], Syed G.A. Moinuddin[4], Christopher C. Azubuike[1], Delyana P. Vasileva[1], Dawn M. Klingeman[1], Leah H. Hochanadel[1], Alexander R. Fisch[1], Brian C. Sanders[1], Lindsay D. Eltis[5], Richard J. Giannone[1,3], Laurence B. Davin[4], Norman G. Lewis[4], John Ralph[2,6], James G. Elkins[1,3], Joshua K. Michener[1,3,†]

[1]Biosciences Division, Oak Ridge National Laboratory, Oak Ridge, TN, 37830
[2]Great Lakes Bioenergy Research Center, Wisconsin Energy Institute, University of Wisconsin, Madison, WI, 53726
[3]Center for Bioenergy Innovation, Oak Ridge National Laboratory, Oak Ridge, TN, 37830.
[4]Institute of Biological Chemistry, Washington State University, Pullman, WA 99164
[5]Department of Microbiology and Immunology, The University of British Columbia, Vancouver, British Columbia, V6T 1Z3
[6]Department of Biochemistry, University of Wisconsin, Madison, WI, 53726

[‡]Present address: Department of Wood Science, Oregon State University, Corvallis, OR

[†]To whom correspondence should be addressed: michenerjk@ornl.gov

Supplemental Methods:

Synthesis of authentic analytical standards

Compound **1**, Lariciresinoic acid, *4-(4-hydroxy-3-methoxybenzyl)-2-(4-hydroxy-3-methoxyphenyl)tetrahydrofuran-3-carboxylic acid*

Compound **1** was synthesized by selective hydrogenolysis of compound **2**, 3,6-bis(4-hydroxy-3-methoxyphenyl)tetrahydro-1H,3H-furo[3,4-c]furan-1-one, which was made from cross-coupling of ferulic acid and coniferyl alcohol according to a published method (1).

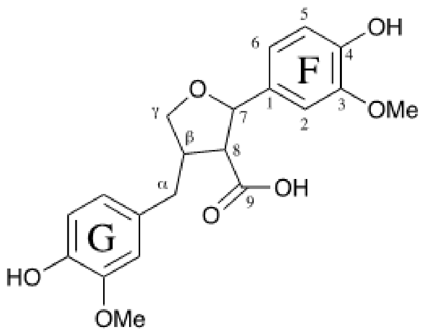

Briefly, compound **2** (5 mg) was dissolved in 3 mL aqueous methanol (MeOH/H_2O, 2/1 v/v), to which 2 mg $PdCl_2$ and 100 mg ammonium formate were added. While stirring the mixture, 2 mL of 0.5 M NaOH was slowly added (over 10 min) via a syringe. The mixture was stirred for 2 h. The mixture was acidified by adding 1.5 mL 1 M HCl solution and diluted with 30 mL water. The crude products were recovered by extraction with ethyl acetate (30 mL). The ethyl acetate solution was evaporated on a rotary evaporator under reduced pressure, producing the crude product as an oil that was purified by thin-layer chromatography (TLC, 0.25 mm silica). About 1 mg of **1** was obtained. NMR (700 MHz, acetone-d_6, Figures S15-S16): δ_H 6.96 (1H, d, J = 1.9 Hz, F2), 6.85 (1H, d, J = 2.0 Hz, G2), 6.80 (1H, dd, J = 8.1, 1.9 Hz, F6), 6.96 (1H, d, J = 8.1 Hz, F5), 6.85 (1H, d, J = 8.0 Hz, G5), 6.67 (1H, dd, J = 8.0, 2.0 Hz, G6), 5.13 (1H, d, J = 7.2 Hz, F7), 4.00 (1H, dd, J = 8.4, 6.3 Hz, Gγ2), 3.83 (3H, s, G3-OMe), 3.81 (3H, s, F3-OMe), 3.72 (1H, dd, J = 8.5, 6.8 Hz, Gγ1), 3.06 (1H, dd, J = 8.6, 7.2 Hz, F8), 2.93 (1H, m, Gβ), 2.85 (1H, dd, J = 13.5, 5.0 Hz, Gα2), 2.85 (1H, dd, J = 13.5, 10.9 Hz, Gα1); δ_C 173.5 (F9), 148.2 (G3), 148.2 (F3), 146.8 (F4), 145.8 (G4), 134.8 (F1), 132.1 (G1), 121.9 (G6), 119.4 (F6), 115.7 (G5), 115.4 (F5), 113.0 (G2), 110.1 (F2), 83.3 (F7), 73.2 (Gγ), 56.8 (F8), 56.1 (G3-OMe + F3-OMe), 44.9 (Gβ), 35.0 (Gα).

Compound **3**, (±)-Imperanene, *(E)-4,4'-(3-(hydroxymethyl)but-1-ene-1,4-diyl)bis(2-methoxyphenol)*

Compound **3**, racemic imperanene, was synthesized from ferulic acid dilactone in about 40% overall yield, via alkaline hydrolysis, intramolecular lactonization, hydrogenation, decarboxylation, acylation, and reduction. The detailed procedures will be published separately. NMR (500 MHz, acetone-d_6): δ_H 6.98 (d, J = 1.89 Hz, 1H, B2), 6.82 (d, J = 1.86 Hz, 1H, A2), 6.79 (dd, J = 8.20, 1.86 Hz, 1H, B6), 6.73 (d, J = 8.1 Hz, 1H), 6.70 (d, J = 8.0 Hz, 1H), 6.65 (dd, J = 8.0, 1.9 Hz, 1H, A6), 6.25 (d, J = 15.9 Hz, 1H, Bα), 6.05 (dd, J = 15.9, 8.2 Hz, 1H, Bβ), 3.82 (s, 3H, B-OMe), 3.76 (s, 3H, A-OMe), 3.55 (dd, J = 9.2, 4.6 Hz, 2H, Aγ), 3.54 (dd, J = 13.0, 5.65 Hz, 1H, Aα1), 2.62– 2.52 (m, 2H, Aα2 + Aβ); δ_C 148.3 (B3), 147.9 (A3), 146.8 (B4), 145.4 (A4), 132.6 (A1), 131.6 (Bα), 130.8 (B1), 130.0 (Bβ),

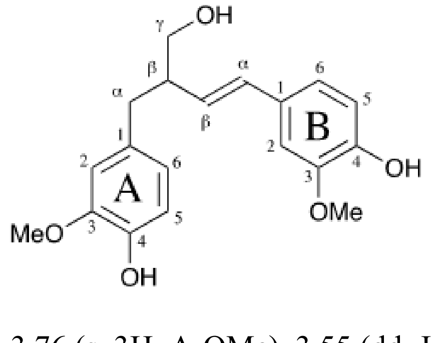

122.5 (A6), 120.1 (B6), 115.7 (B5), 115.3 (A5), 113.7 (A2), 109.8 (B2), 65.6 (Aγ), 56.06, 56.08, 48.8 (Aβ), 38.2 (Aα).

The NMR data for the obtained compound **3** were consistent with those published (2).

Compound **4**, Diguaiacylbutadiene (DGBD), *4,4'-((1E,3E)-buta-1,3-diene-1,4-diyl)bis(2-methoxyphenol)*

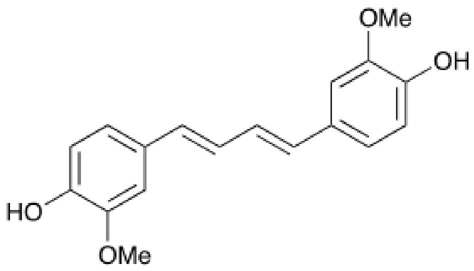

Compound **4** was synthesized by alkaline hydrolytic decarboxylation of ferulic acid dilactone that was obtained from peroxidase-catalyzed free-radical coupling of ferulic acid in aqueous acetone media (3). Briefly, 1.0 g ferulic acid dilactone was dissolved in 10 mL conc. ammonia solution (24%). The solution was capped in a 25 mL hydrolytic bottle and kept in a 90 °C oven overnight (16 h). Once the bottle was cooled to room temperature in a fume hood, the solution was transferred to a 200 mL round-bottom flask. The ammonia was removed by evaporation on a rotary evaporator under reduced pressure. The concentrated residues were diluted with water and acidified by adding 1 M HCl solution and the crude products were recovered by ethyl acetate extraction. Once dried over anhydrous $MgSO_4$, the ethyl acetate solution was filtered to remove the inorganics and evaporated to produce an oily crude product that was further fractionated by flash-column chromatography on silica. Compound **4** was in the first eluted fraction, 54 mg (5% yield). The NMR data for compound **4** were consistent with those published in the literature (4).

NMR characterization of synthesized standards

NMR spectra of synthesized standards were acquired on a Bruker Biospin (Billerica, MA) Avance III 500 MHz spectrometer equipped with a 5-mm TCI $^{1}H/^{13}C/^{15}N$ cryoprobe. The central acetone peaks (δ_C 29.8, δ_H 2.04 ppm) were used as the internal references. The usual array of NMR experiments [1D ^{1}H, 1D ^{13}C, 2D COSY, 2D HSQC, and 2D HMBC] was used to authenticate assignments made below.

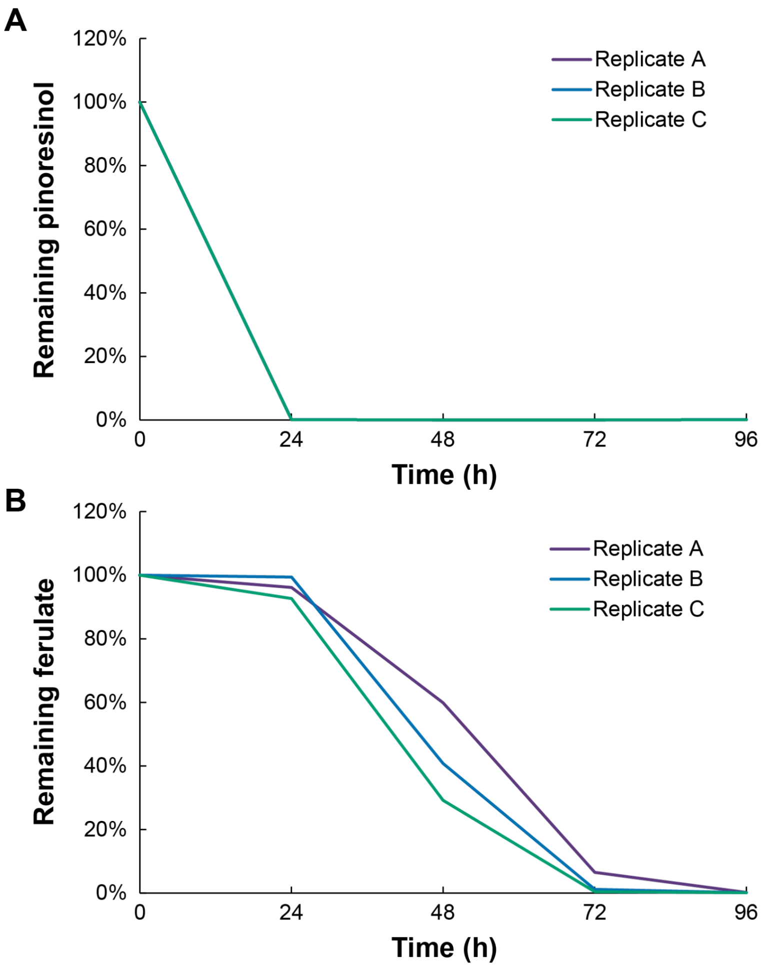

Figure S1. Substrate consumption during growth of *Novosphingobium rhizosphaerae* sp. LY with (A) (+)-pinoresinol or (B) ferulate as a sole growth substrate. Cultures were grown at 30 °C in 30 mL of liquid minimal medium 457 with 0.1 g/L of (+)-pinoresinol or 1 g/L of ferulate as the sole growth substrate. Samples were taken every 24 h, filter sterilized, and analyzed by LC-MS/MS to quantify residual substrate. Three biological replicates are shown for each condition; in Figure S1A, the three replicates are indistinguishable by eye.

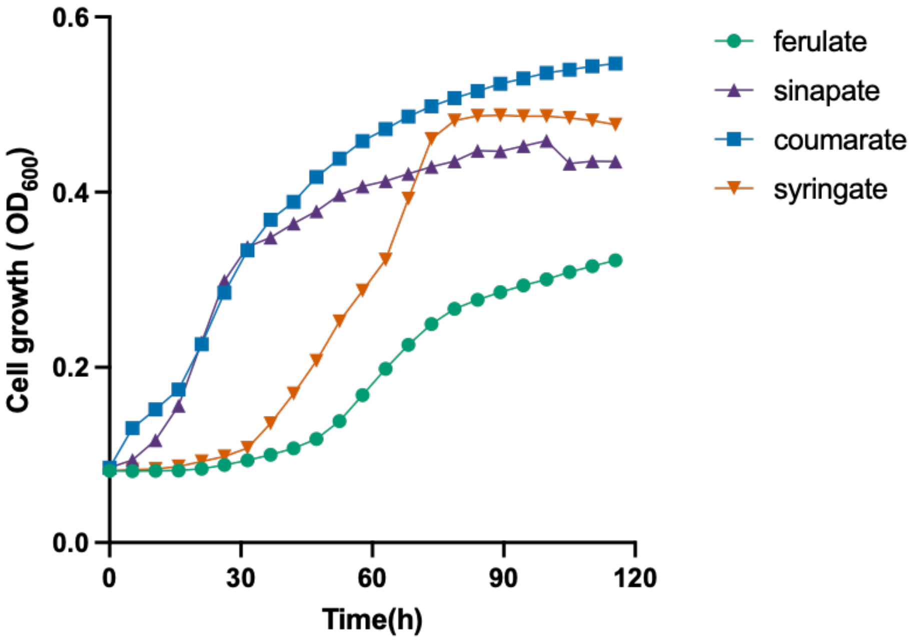

Figure S2. Growth of *Novosphingobium rhizosphaerae* sp. LY with indicated lignin-derived aromatic monomers as a sole carbon source. Data presented are the means of three independent experiments. Growth substrates were added to a final concentration of 1 g/L and cultures were incubated at 30 °C.

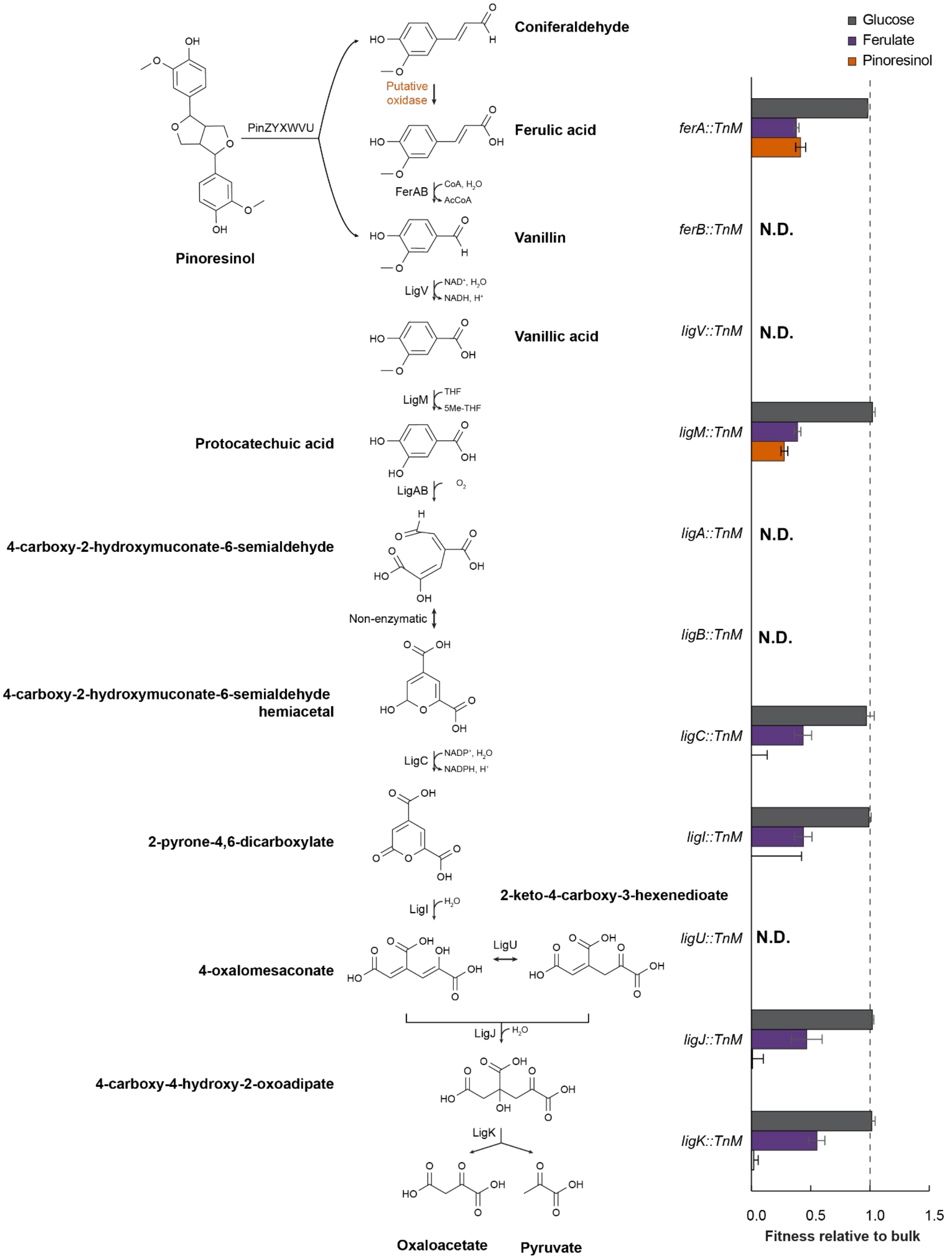

Figure S3. Predicted pathway for catabolism of coniferaldehyde, ferulate, and vanillin in LY. Predicted genes in LY and the corresponding SYK-6 homologs are shown in Table S1. Error bars show one standard deviation, calculated from three biological replicates. N.D.: Not determined; disruptions in *ferB*, *ligV, ligA*, *ligB*, and *ligU* were identified in the initial barcode mapping but not in the time-zero samples used for the fitness experiments.

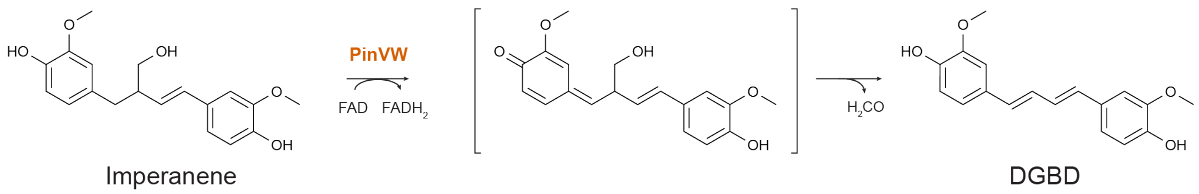

Figure S4. Proposed mechanism for the PinVW-catalyzed deformylation of imperanene. The quinone methide intermediate is similar to that formed during deformylation of diguaiacylpropanediol (DGPD) by LdpA (5).

A) **DGBD: experimental sample**

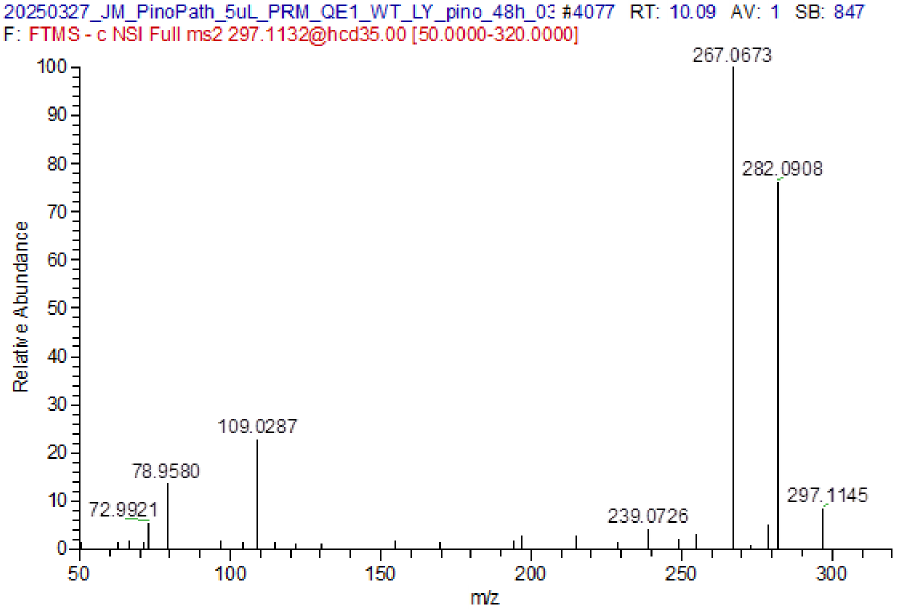

B) **Diguaiacylbutadiene (DGBD): standard**

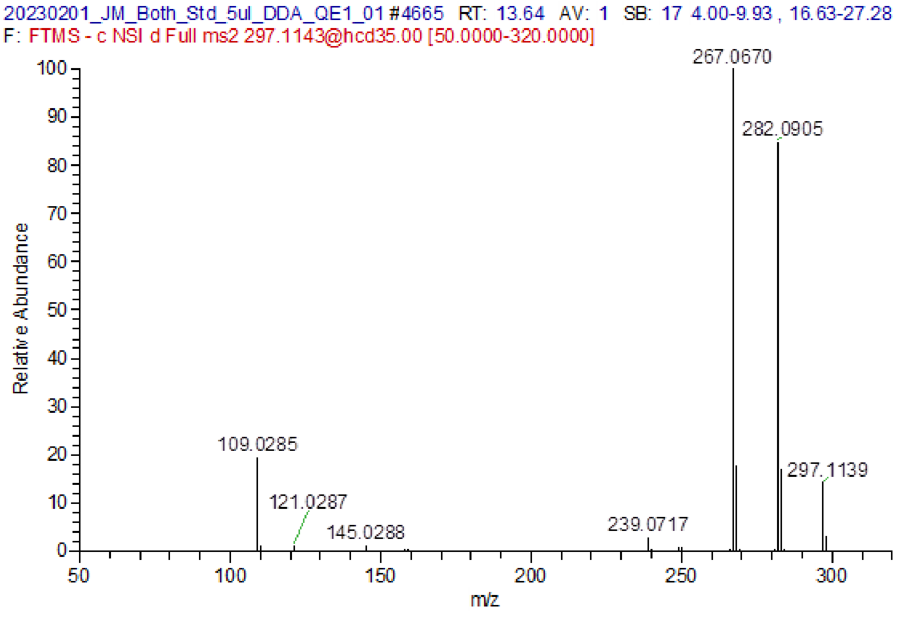

Figure S5. Mass spectra of DGBD. (A) Experimental mass spectrum of DGBD isolated from JMN139 after incubation with (+)-pinoresinol. (B) Mass spectrum of chemically-synthesized DGBD.

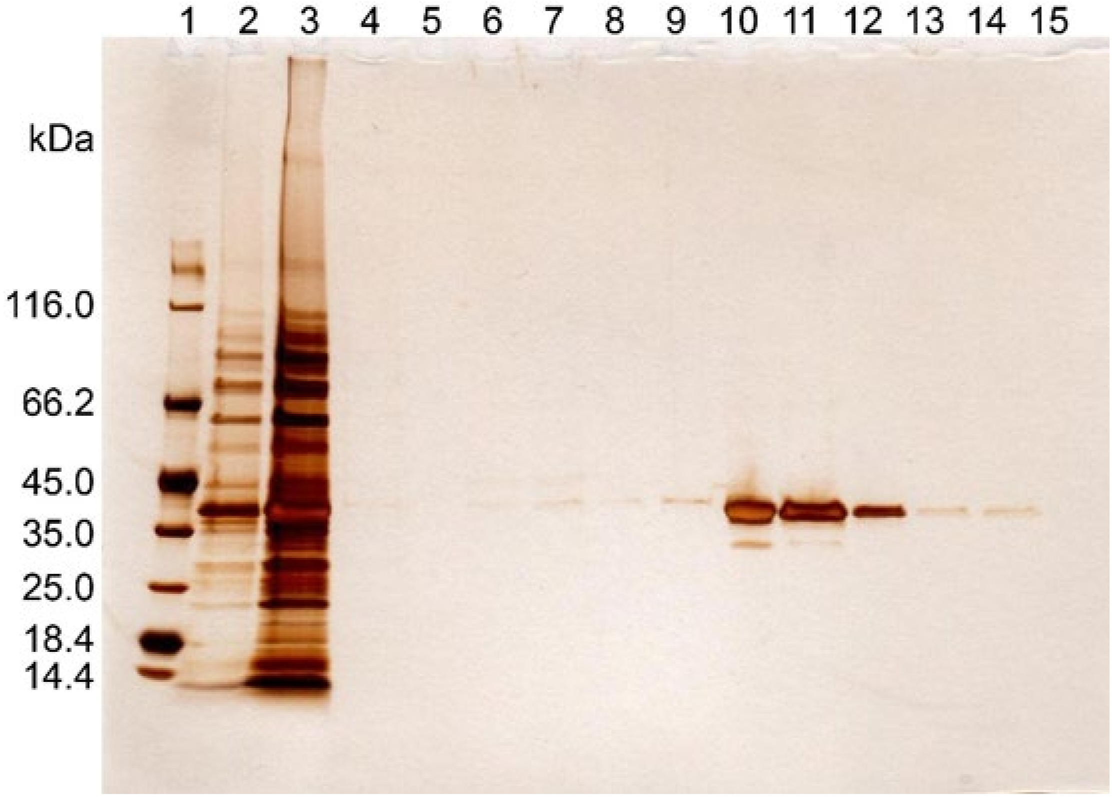

Figure S6. Heterologous expression and purification of PinZ. PinZ was purified to apparent homogeneity after metal chelate affinity chromatography. Lane 1, MW protein standards. Lane 2, flow through from the metal chelate affinity chromatography column. Lanes 3 to 5, fractions eluted with binding buffer to remove unbound proteins. Lanes 6 to 9, fractions eluted with binding buffer containing 15 mM imidazole. Lanes 10 to 13, fractions eluted with binding buffer containing 100 mM imidazole. Lanes 14 and 15, fractions eluted with binding buffer containing 300 mM imidazole. PinZ, shown in Lanes 10 and 11 was used for enzyme assays.

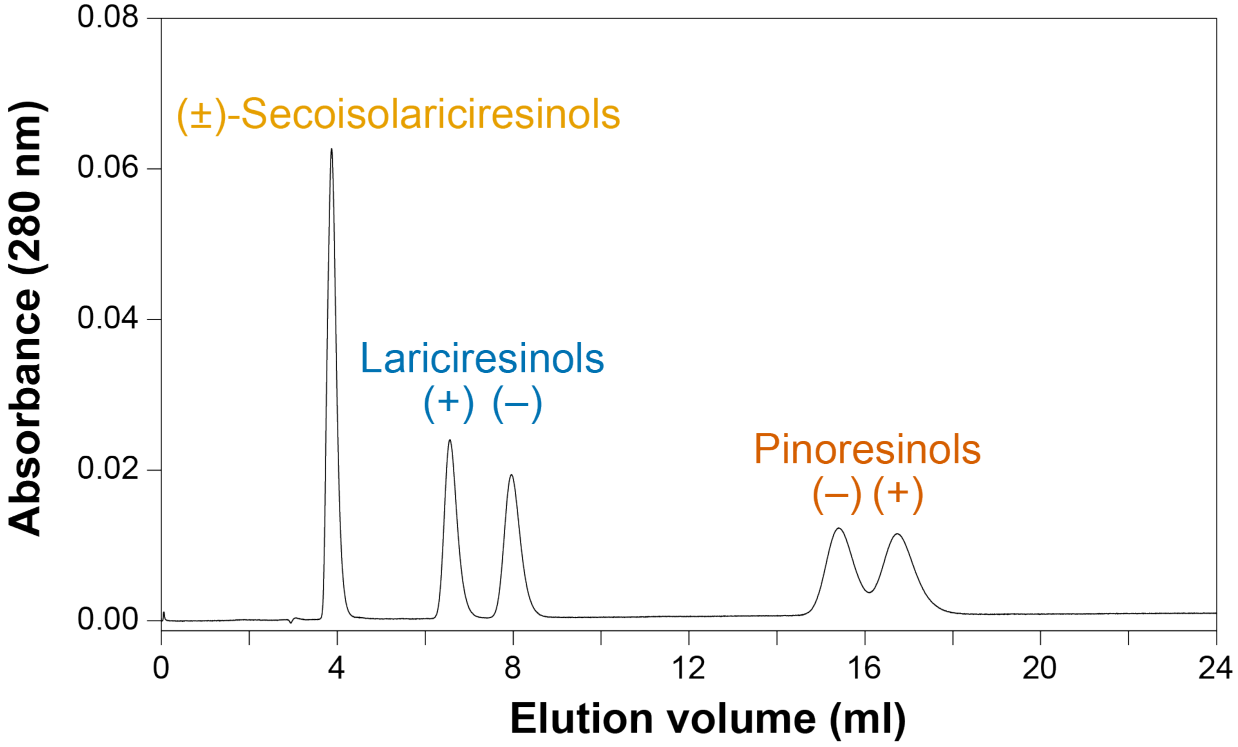

Figure S7. Chiral HPLC analysis of synthesized standards. Enantiomers of authentic (racemic) standards of pinoresinol and lariciresinol were well-resolved using chiral-phase HPLC (Chiralcel OC column), whereas secoisolariciresinol enantiomers could not be separated using these conditions.

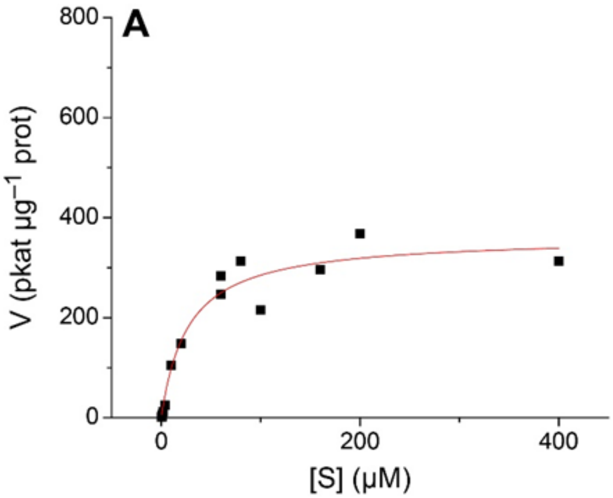

Figure S8. Michaelis-Menten plots for PinZ with (±)-pinoresinols (A) and (+)-pinoresinol (B).

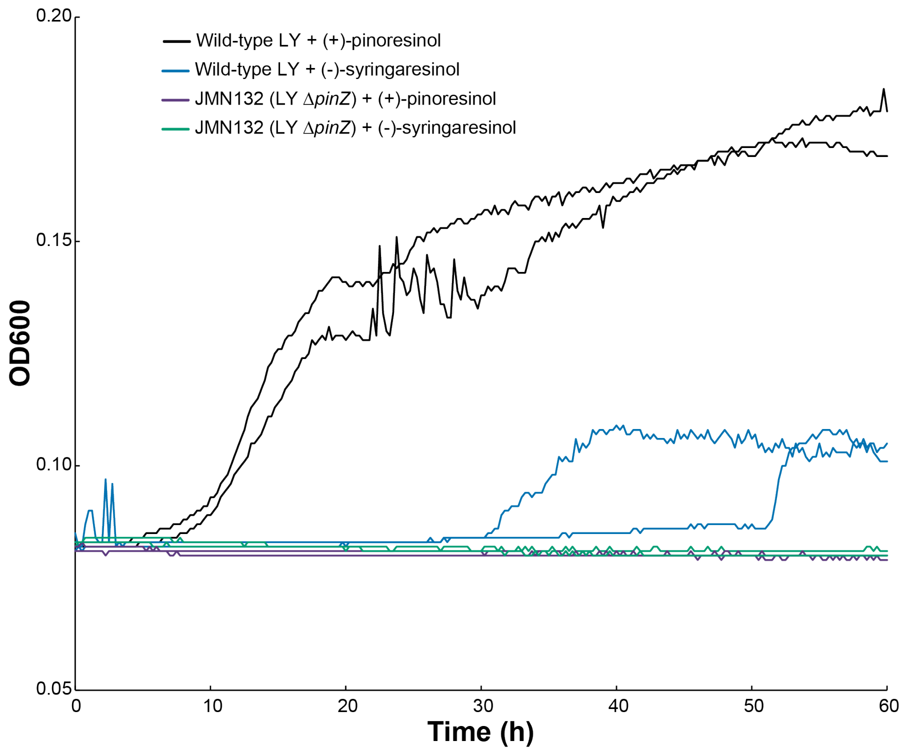

Figure S9. Growth of wild-type and Δ*pinZ N. rhizosphaerae* LY with (+)-pinoresinol and (-)-syringaresinol. Carbon sources were added to a final concentration of 0.1 g/L and cultures were maintained at 30 °C with shaking in a 48-well plate. Two replicates are shown for each combination of strain and growth substrate.

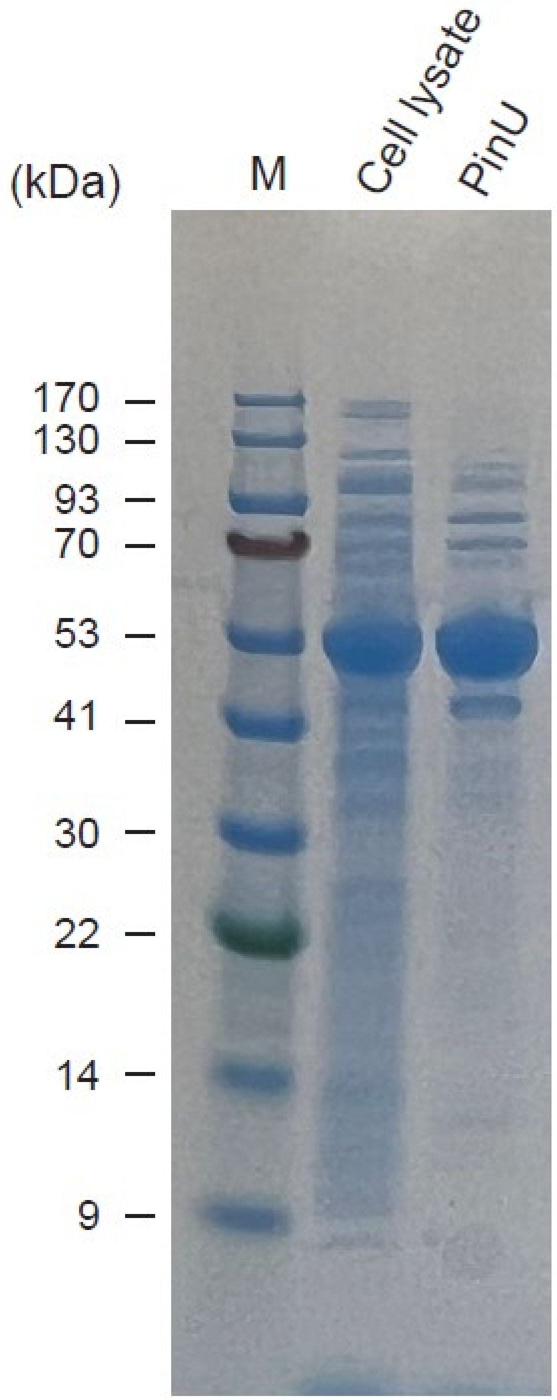

Figure S10. Heterologous expression and purification of PinU. Recombinant PinU was expressed from pET-24a(+) in *E. coli* BL21(DE3) and partially purified using ion-exchange chromatography. Each lane contains 20 µg of protein. M, protein marker.

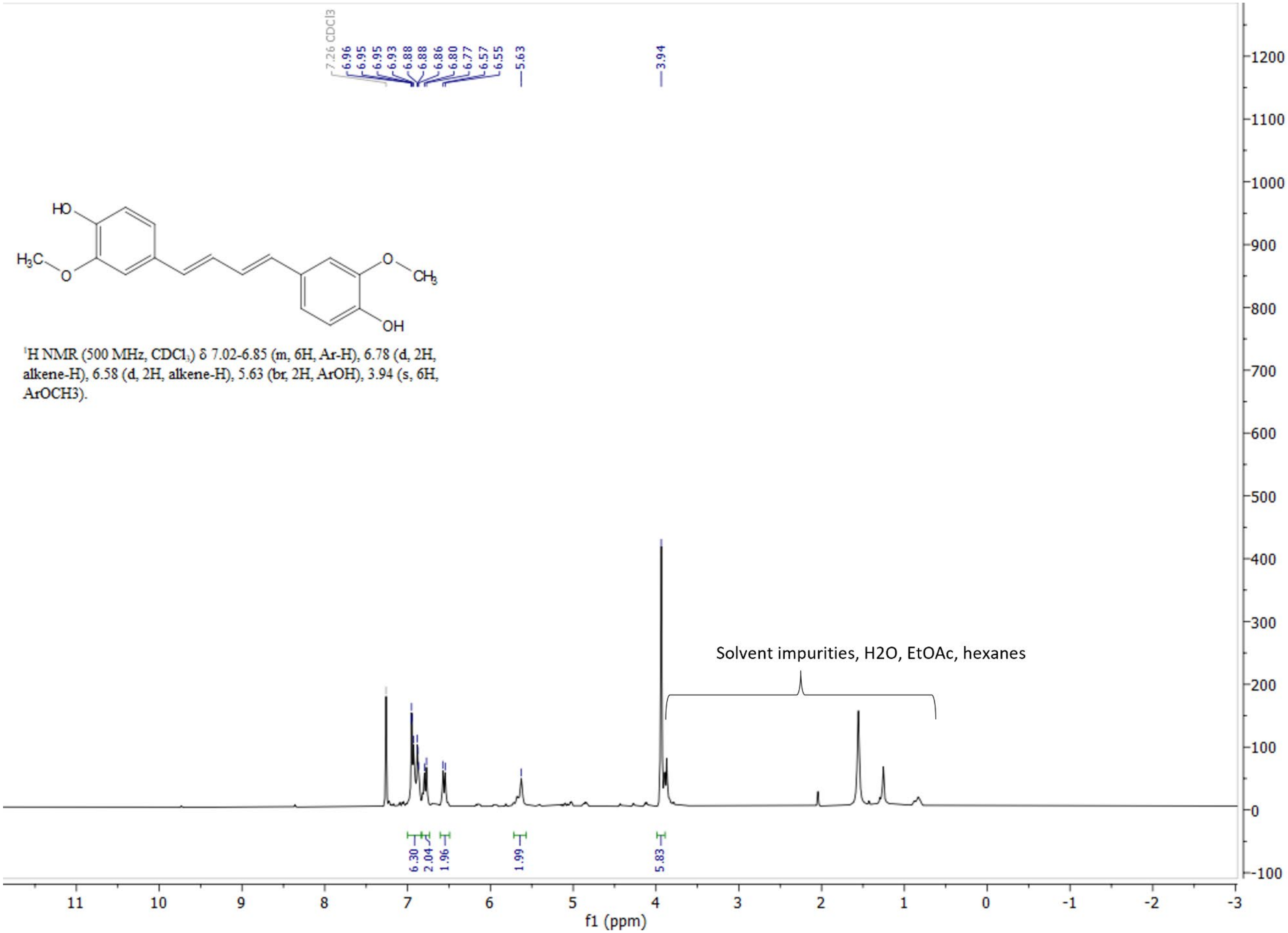

Figure S11: ^{1}H NMR spectrum of isolated Compound 4, diguaiacylbutadiene (DGBD)

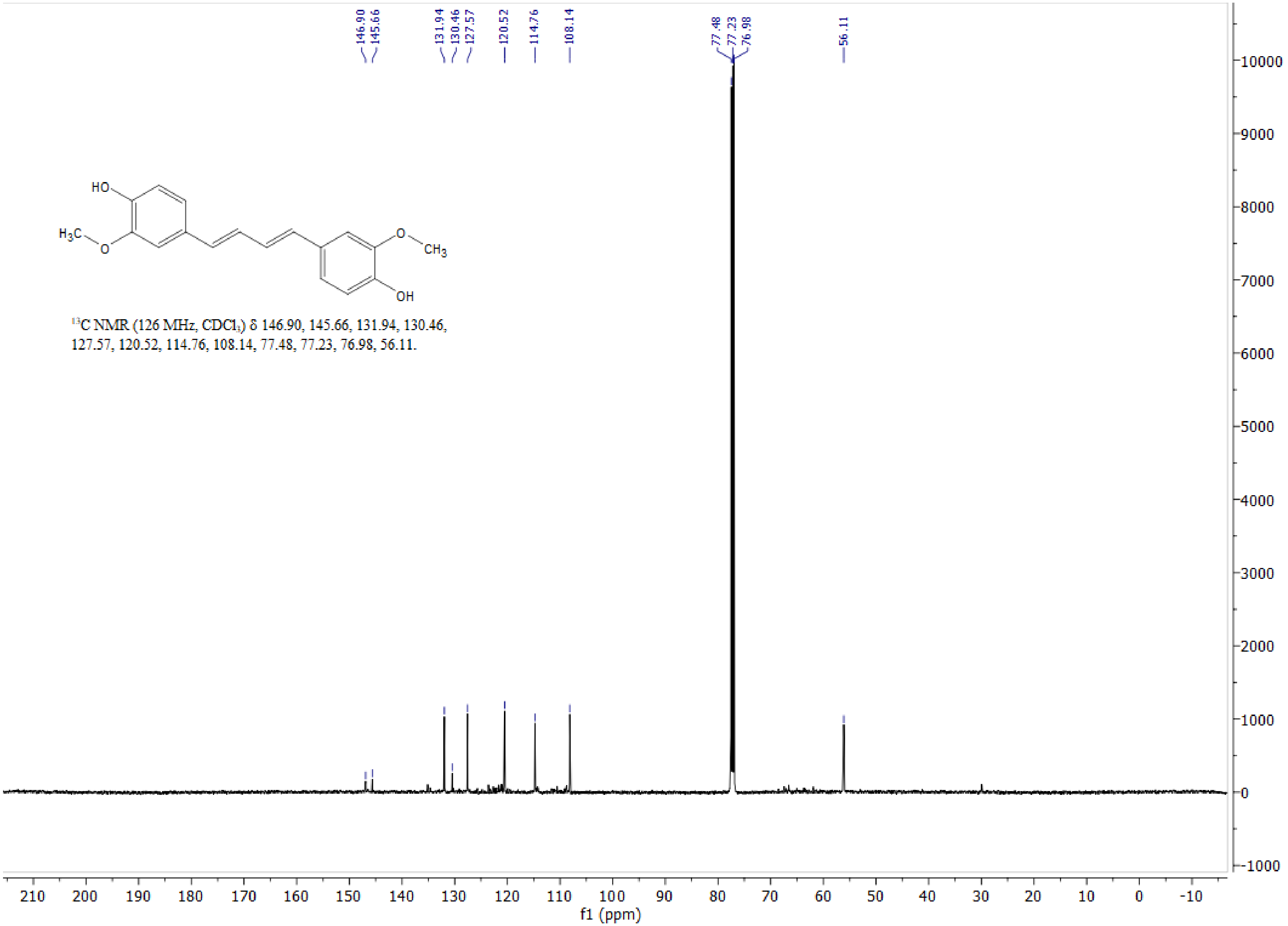

Figure S12: ^{13}C NMR spectrum of isolated Compound 4, diguaiacylbutadiene (DGBD)

A) **Vanillin: experimental sample**

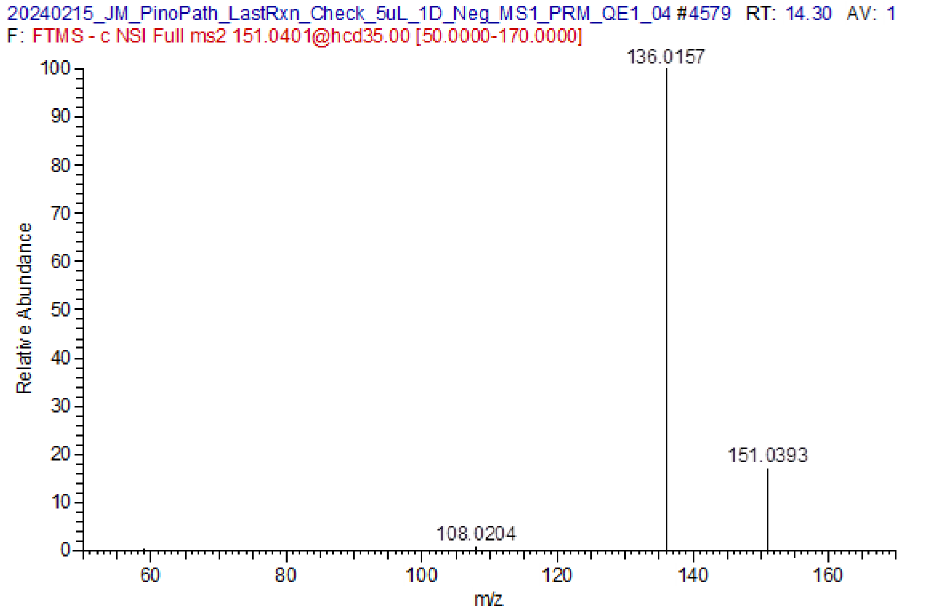

B) **Vanillin: Mass Bank of America reference**
https://mona.fiehnlab.ucdavis.edu/spectra/display/MoNA038047

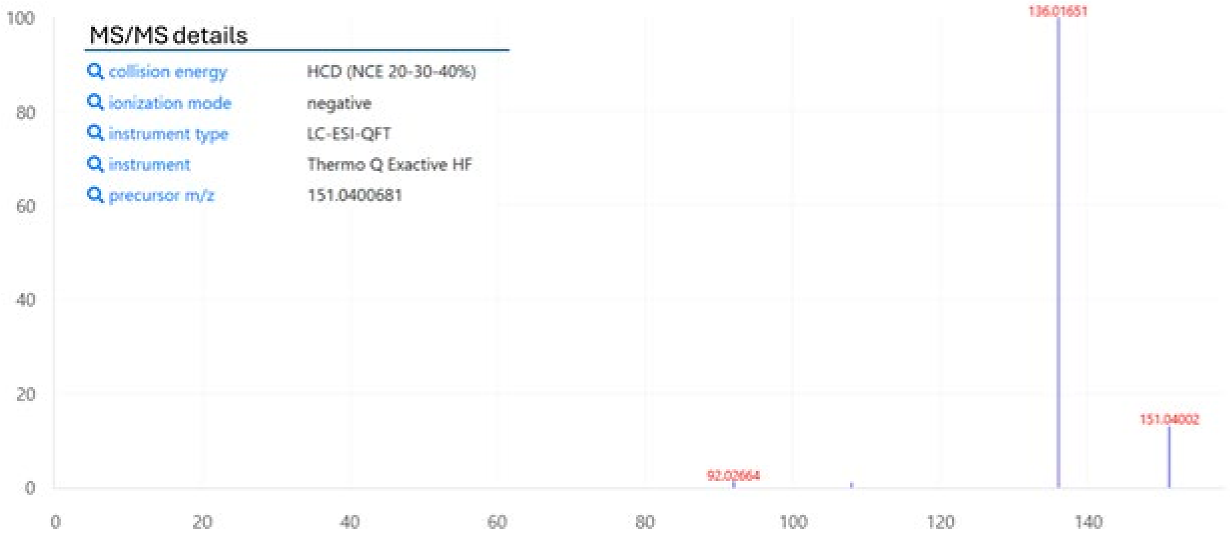

Figure S13: Mass spectrum of vanillin. (A) Experimental mass spectrum of the vanillin peak after incubation of DGBD with PinU. (B) Reference mass spectrum of vanillin

A) Coniferaldehyde experimental sample

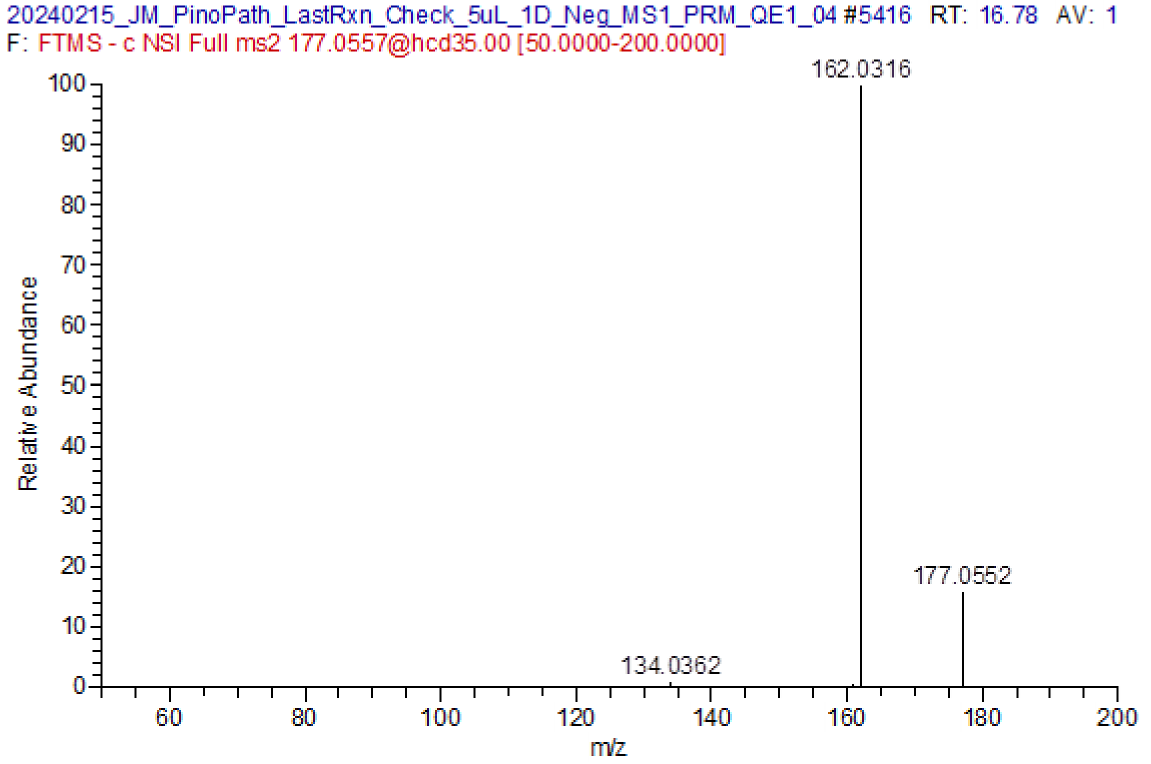

B) Coniferaldehyde: Mass Bank of America reference spectrum
https://mona.fiehnlab.ucdavis.edu/spectra/display/FiehnHILIC001128

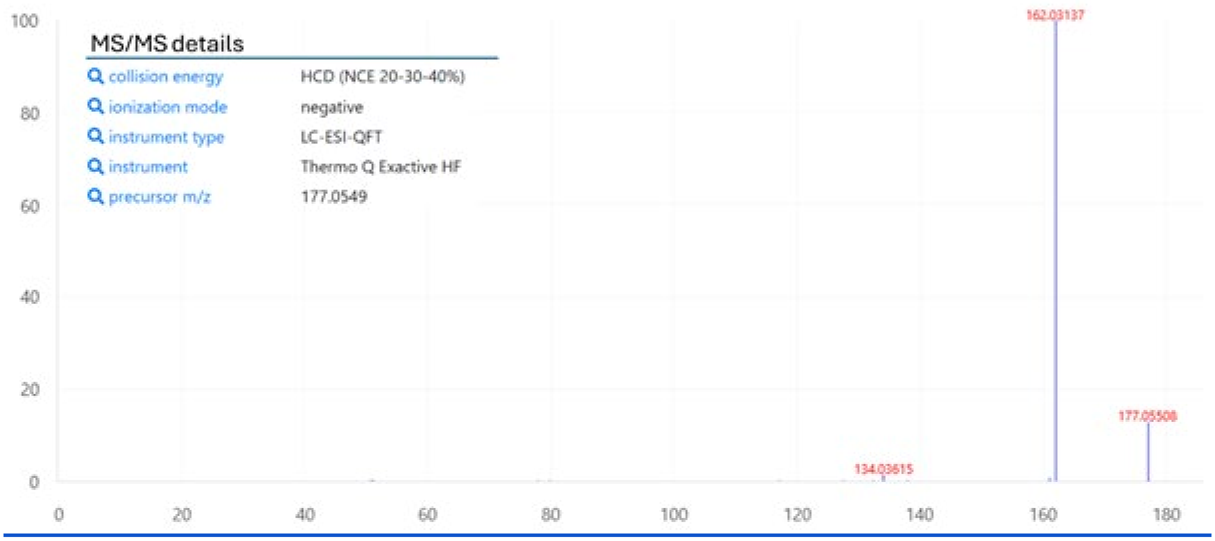

Figure S14. Mass spectra of coniferaldehyde. (A) Experimental mass spectrum of the coniferaldehyde peak after incubation of DGBD with PinU. (B) Reference mass spectrum of coniferaldehyde

Flw10-T, TLC-purified product, "**Lariciresinoic acid**"
PROTON, Acetone-d6

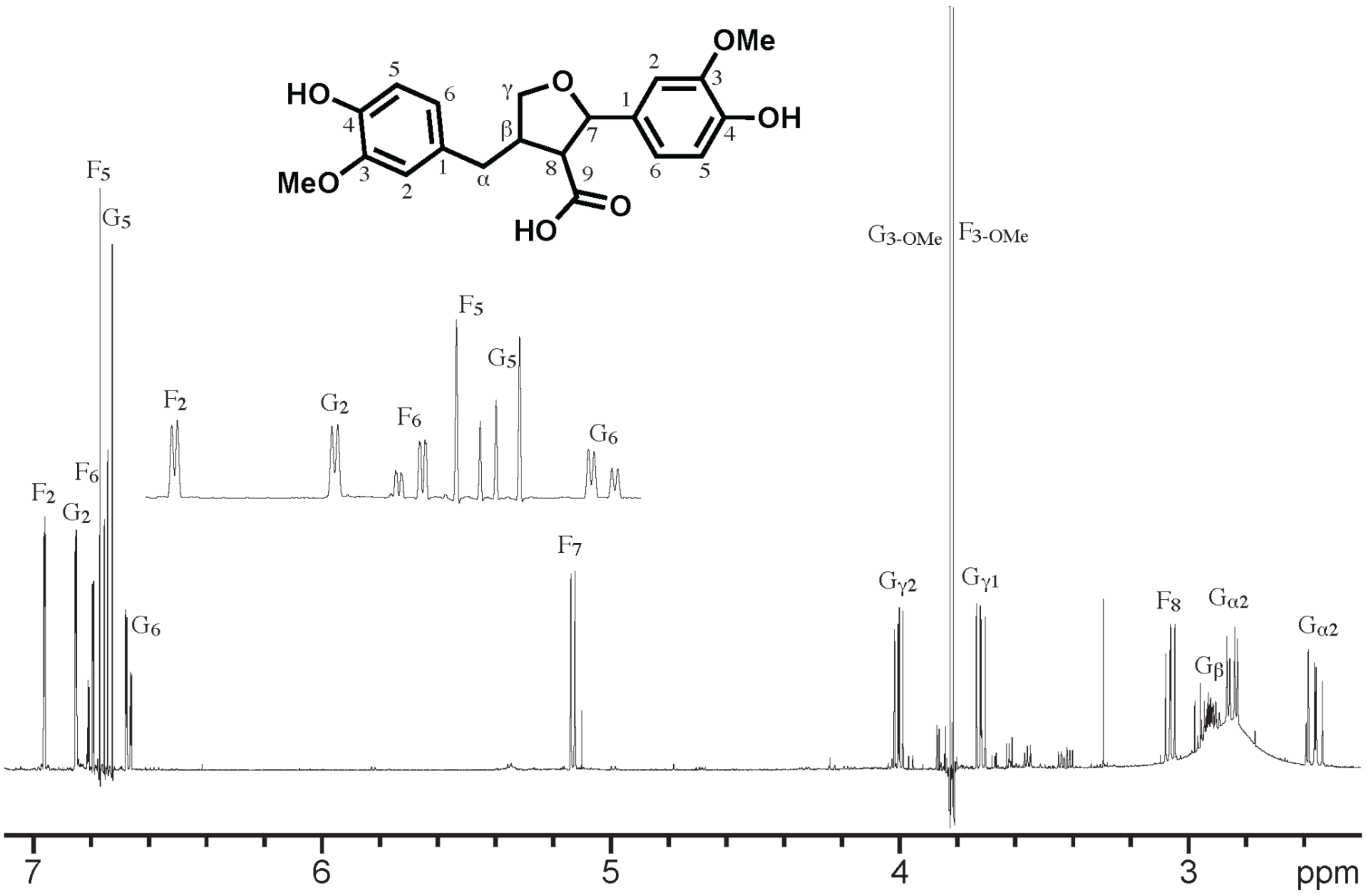

Figure S15. ^{1}H NMR spectrum of synthesized compound 1, lariciresinoic acid

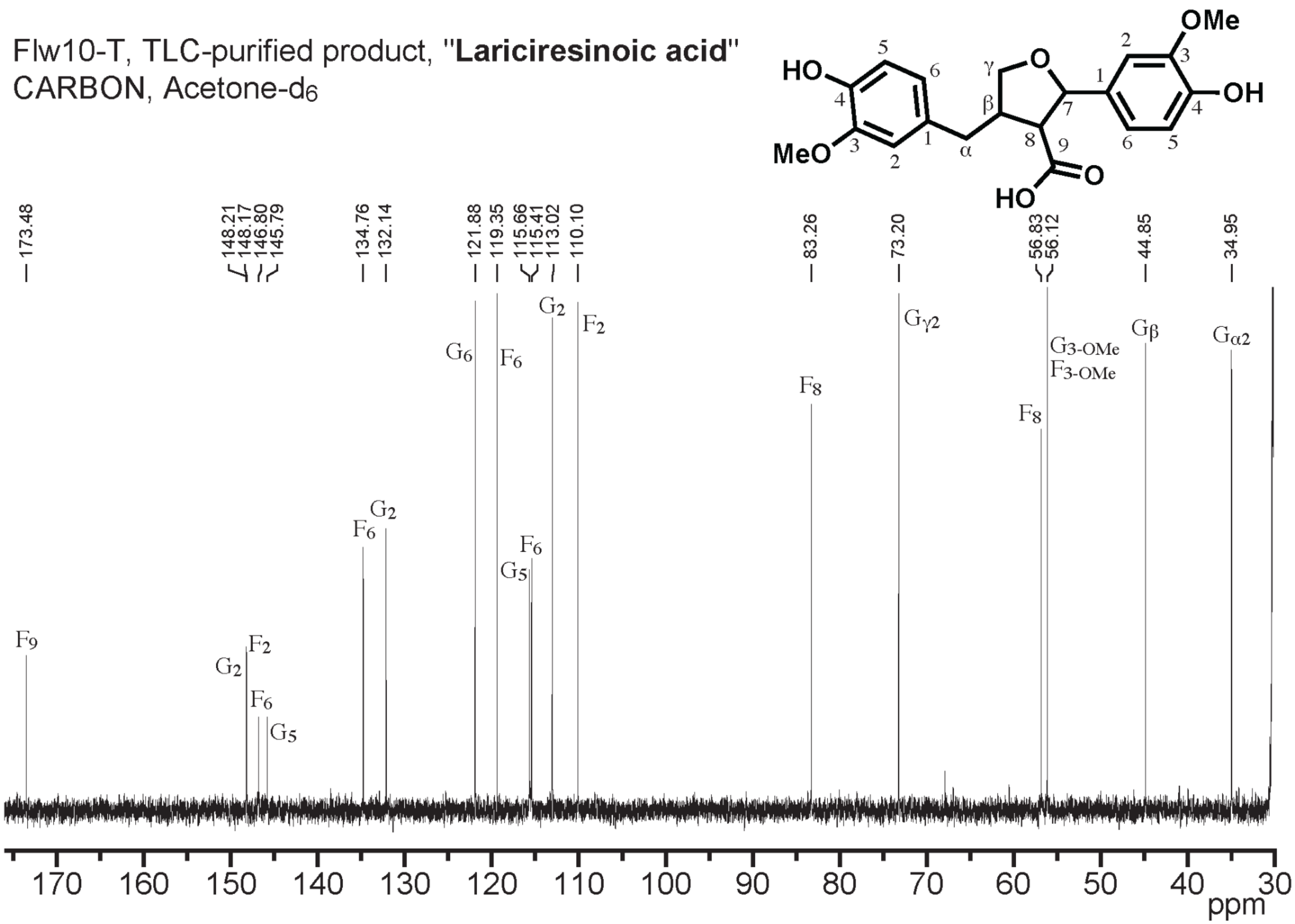

Figure S16. ^{13}C NMR spectrum of synthesized compound 1, lariciresinoic acid.

Table S1. Homologs of lignin catabolism genes from *S. lignivorans* SYK-6 found in *N. rhizosphaerae* LY

Gene name	Annotation	SYK-6 locus tag	LY homolog locus tag	% amino acid identity	Fitness effect with pinoresinol	Fitness effect with ferulate	Fitness effect with glucose
GGE (β–*O*–4) catabolism							
ligD	$NAD(P)^+$-dependent dehydrogenase (short-chain alcohol dehydrogenase family)	SLG_08640	None found	NA			
ligE	β-Etherase	SLG_08660	R9J51_10665	63.2	0.96±0.05	0.97±0.02	0.98±0.03
ligF	Glutathione *S*-transferase family protein	SLG_08650	R9J51_11700	64.0	1.01±0.09	1.02±0.12	1.04±0.02
ligG	Glutathione *S*-transferase	SLG_08670	None found	NA			
ligL	$NAD(P)^+$-dependent dehydrogenase (short-subunit alcohol dehydrogenase family)	SLG_33660	None found	NA			
ligN	$NAD(P)^+$-dependent dehydrogenase (short-subunit alcohol dehydrogenase family)	SLG_35900	None found	NA			
ligO	NADP-dependent 3-hydroxy acid dehydrogenase YdfG	SLG_35880	None found	NA			

ligP	β-Etherase	SLG_32600	R9J51_10665	71.6	0.96±0.05	0.97±0.02	0.98±0.03
ligQ	Glutathione-dependent disulfide-bond oxidoreductase	SLG_04120	R9J51_19385	63.8	N.D.	N.D.	N.D.
hpvZ	HPV oxidase	SLG_12830	None found	NA			
DDVA (5–5′) catabolism							
ddvK	DDVA MFS family permease	SLG_07710	None found	NA			
ddvR	MarR family transcriptional regulator	SLG_07780	None found	NA			
ddvT	DDVA TonB-dependent transporter	SLG_07650	None found	NA			
ligW2	5-Carboxyvanillate decarboxylase	SLG_12810	R9J51_00160	68.5	0.99±0.10	1.01±0.05	1.01±0.05
ligW	5-Carboxyvanillate decarboxylase	SLG_07850	None found	NA			
ligXa	5,5′-Dehydrodivanillate *O*-demethylase oxygenase subunit	SLG_07770	None found	NA			
ligXc	5,5′-Dehydrodivanillate *O*-demethylase ferredoxin subunit	SLG_08500	R9J51_18420	63.2	N.D.	N.D.	N.D.

ligXd	5,5′-Dehydrodivanillate *O*-demethylase ferredoxin reductase subunit	SLG_21200	R9J51_03850	65.9	0.33±0.10	1.13±0.03	0..97±0.02
ligY	OH-DDVA meta-cleavage compound hydrolase	SLG_07750	None found	NA			
ligZ	OH-DDVA oxygenase	SLG_07720	None found	NA			
Phenylcoumaran (β–5)							
bzaA	Aromatic aldehyde dehydrogenase	SLG_27910	R9J51_21665	56.6	0.86±0.04	0.89±0.03	0.95±0.02
phcC	DCA-C oxidase	SLG_09480	None found	NA			
phcD	DCA-C oxidase	SLG_09500	None found	NA			
phcF	DCA-CC decarboxylase	SLG_09360	None found	NA			
phcG	DCA-CC decarboxylase	SLG_09370	R9J51_15745	71.3	1.07±0.04	1.06±0.01	1.03±0.01
Pinoresinol (β–β): Homologs in SYK-6 of genes in LY							
pinZ	Pinoresinol reductase	SLG_07320	R9J51_00450	73.3	0.60±0.09	0.97±0.19	1.01±0.06
pinY	Lariciresinol oxidase	None found	R9J51_15355	NA	0.44±0.33	0.96±0.17	0.94±0.06

pinX	Lariciresinoate decarboxylase	None found	R9J51_00385	NA	0.36±0.05	1.02±0.08	0.97±0.01
pinW	Imperanene oxidase	None found	R9J51_00375	NA	0.48±0.24	0.97±0.12	1.02±0.03
pinV	Cytochrome c_6	None found	R9J51_00370	NA	0.25±0.30	1.07±0.07	1.01±0.08
pinU	Diguaiacylbutadiene dioxygenase	None found	R9J51_00455	NA	N.D.	N.D.	N.D.
Phenylpropanoid chain metabolism							
ferA	Feruloyl-CoA ligase	SLG_25020	R9J51_02980	70.2	0.41±0.04	0.38±0.02	0.98±0.002
ferB	*p*-Hydroxycinnamoyl CoA hydratase/lyase	SLG_25030	R9J51_10560	72.3	N.D.	N.D.	N.D.
ferC	MarR family transcriptional regulator	SLG_25040	None found	N/A			
desV	Aromatic aldehyde dehydrogenase	SLG_28320	R9J51_00415	57.1	0.93±0.03	1.04±0.01	1.00±0.02
ligV	Vanillin dehydrogenase	SLG_07060	R9J51_10555	77.7	N.D.	N.D.	N.D.
O-Demethylation and downstream C1 metabolism							
desA	Syringate *O*-demethylase	SLG_25000	R9J51_10670	71.9	0.88±0.08	1.00±0.21	0.95±0.03

desR	MarR family transcriptional regulator	SLG_12870	R9J51_00135	56.5	N.D.	N.D.	N.D.
ligH	Formate-tetrahydrofolate ligase	SLG_12760	None found	NA			
ligM	Aminomethyl transferase family protein	SLG_12740	R9J51_00310	76.9	0.28±0.03	0.39±0.03	1.02±0.02
metF	Methylenetetrahydrofolate reductase	SLG_12750	R9J51_00305	59.3	1.14±0.06	1.23±0.06	1.09±0.06
Ring cleaving pathways							
desB	Gallate dioxygenase	SLG_03330	None found	N/A			
desX	IclR family transcriptional repressor	SLG_24970	R9J51_10630	57.7	0.35±0.40	0.87±0.14	1.06±0.04
desZ	3-*O*-Methylgallate 3,4-dioxygenase	SLG_19030	None found	N/A			
ligA	Protocatechuate 4,5-dioxygenase, α subunit	SLG_12510	R9J51_00480	65.8	N.D.	N.D.	N.D.
ligB	protocatechuate 4,5-dioxygenase, β subunit	SLG_12500	R9J51_00475	70.1	N.D.	N.D.	N.D.
ligC	4-Carboxy-2-hydroxymuconate-6-semialdehyde dehydrogenase	SLG_12490	R9J51_00470	82.6	0.00±0.13	0.44±0.07	0.97±0.07

ligI	2-Pyrone-4,6-dicarboxylate hydrolase	SLG_12570	R9J51_00510	79.1	0.00±0.42	0.44±0.07	0.99±0.02
ligJ	2-Keto-4-carboxy-3-hexenedioate hydratase	SLG_12520	R9J51_00485	83.9	0.01±0.09	0.47±0.13	1.02±0.01
ligK	4-Carboxy-4-hydroxy-2-oxoadipate aldolase	SLG_12550	R9J51_00500	80.3	0.02±0.04	0.55±0.07	1.01±0.03
ligR	LysR family transcriptional regulator	SLG_12540	R9J51_00495	59.8	N.D.	N.D.	N.D.
ligU	4-Oxalomesaconate tautomerase	SLG_12560	R9J51_00505	77.0	N.D.	N.D.	N.D.
Lignostilbene dioxygenases: eight homologs are present in SYK-6, two in LY							
lsdA	Carotenoid oxygenase family protein	SLG_12580	R9J51_00455	61.4	N.D.	N.D.	N.D.
lsdB	Carotenoid oxygenase family protein	SLG_09440	R9J51_00455	49.8	N.D.	N.D.	N.D.
lsdC	Carotenoid oxygenase family protein	SLG_11300	R9J51_00140	65.4	1.08±0.02	0.94±0.07	1.05±0.03

lsdD	Carotenoid oxygenase family protein	SLG_12860	R9J51_00140	77.5	1.08±0.02	0.94±0.07	1.05±0.03
lsdE	Carotenoid cleavage dioxygenase	SLG_27300	None found	N/A			
lsdF	Carotenoid oxygenase family protein	SLG_27970	R9J51_00455	62.0	N.D.	N.D.	N.D.
lsdG	Carotenoid oxygenase family protein	SLG_36640	R9J51_00140	64.6	1.08±0.02	0.94±0.07	1.05±0.03
lsdH	Carotenoid oxygenase family protein	SLG_37540	R9J51_00140	62.9	1.08±0.02	0.94±0.07	1.05±0.03

N.A.: Not applicable

Table S2. Amino acid identity of PinU with homologous lignostilbene dioxygenases

**Reference sequence**	**% amino acid identity**
*Na*NOV1	33.6
*Na*NOV2	71.5
*Nr*LsdA	33.1
*Sl*LsdA	53.3
*Sl*LsdB	33.4
*Sl*LsdC	35.2
*Sl*LsdD	35.4
*Sl*LsdE	30.9
*Sl*LsdF	51.7
*Sl*LsdG	36.4
*Sl*LsdH	33.9

Na refers to homologs from *Novosphingobium aromaticivorans* F199. *Sl* refers to homologs from *Sphingobium lignivorans* SYK-6. *Nr* refers to the other lignostilbene dioxygenase homolog in *Novosphingobium rhizosphaerae* LY.

Table S3. Enantiomeric composition (%) of PinZ reactions.

	Pinoresinol		Lariciresinol	
	(+)	(-)	(+)	(-)
**Figure S8**	52	48	50	50
**Figure 4B**	51	49		
**Figure 4C**	52	48	50	50
**Figure 4D**	94	6		
**Figure 4E**	89	11	92	8

Table S4. Kinetic data for *N. rhizosphaerae* PinZ and comparison to published values

Enzyme	Substrate	Organism	K_M (µM)	v_{max} (nkat/mg protein)	k_{cat} (s^{-1})	k_{cat}/K_M (M^{-1} s^{-1})	Reference
**Recombinant proteins**							
NrPinZ	(±)-Pinoresinol	*Novosphingobium rhizosphaerae*	27.0 ± 6.6	361.8 ± 22.4	12.7	4.69×10^5	This work
NrPinZ	(+)-Pinoresinol	*N. rhizosphaerae*	22.1 ± 3.2	307.5 ± 14.6	10.8	4.86×10^5	This work
SlPinZ	(±)-Pinoresinol	*Sphingobium lignivorans*	N.D.	767 ± 50	N.D.	N.D.	(6)
AtPLR1	(+)-Pinoresinol	*A. thaliana*	1.6	0.6	0.14	1.9×10^4	(7)
AtPLR1	(–)-Pinoresinol	*A. thaliana*	7.3	0.6	0.02	1.3×10^4	(7)
PLR_Tp1	(+)-Pinoresinol	*Thuja plicata*	320 ± 2.0	7.9 ± 0.0	0.56	1756	(8)
PLR_Tp1	(–)-Pinoresinol	*T. plicata*	51 ± 9.0	5.8 ± 0.7	0.41	8102	(8)
PLR_Tp2§	(+)-Pinoresinol	*T. plicata*	1.9 ± 0.0	33.0 ± 0.5	2.3	1.19×10^6	(8)
PLR_Tp2§	(–)-Pinoresinol	*T. plicata*	5.2 ± 0.1	12.7 ± 0.1	0.88	1.7×10^5	(8)
**Native proteins**							
PLR_Fi1*	(+)-Pinoresinol	*Forsythia intermedia*	27 ± 1.5	4.5 ± 0.1	0.16	5,820	(9)
PLR_Fi2*	(+)-Pinoresinol	*F. intermedia*	23 ± 1.3	4.8 ± 0.1	0.17	7,280	(9)

N.D.: Not determined

*PLR_Fi1 and PLR_Fi2 were purified (~3000 fold) to homogeneity from ~20 kg *F. intermedia* stems over a month period, hence the lower overall catalytic activity as compared to the recombinant proteins.

§As PLR_Tp2 displayed severe substrate inhibition at high substrate concentrations, the Hill equation was used to obtain kinetic parameters, $S_{0.5}$ instead of K_M and $k_{cat}/S_{0.5}$ instead of k_{cat}/K_m.[8]

Table S5. Strains and plasmids used in this study

Strain	Description	Source
Escherichia coli strains		
WM6026	*lacI*q, *rrnB3*, Δ*lacZ4787*, *hsdR514*, Δ*araBAD567*, Δ*rhaBAD568*, *rph-1*, *att*λ::pAE12(Δ*oriR6K-cat*::Frt5), Δ*endA*::Frt, *uidA*(ΔMluI)::*pir*, *attHK*::pJK1006D(*oriR6K-cat*::Frt5; *trfA*::Frt) *dap*	Lab stock
WM3064	*thrB1004 pro thi rpsL hsdS lacZ*ΔM15 RP4-1360 *Δ(araBAD)567 dapA1341*::[*erm pir(wt)*]	(10)
N. rhizosphaerae sp. LY strains		
LY WT	Pinoresinol-utilizing wild-type strain	This study
JMN128	LY, Δ*R9J51_00455* (*pinX*)	This study
JMN130	LY, Δ*R9J51_00470* (*ligC*)	This study
JMN131	LY, Δ*R9J51_15355* (*pinY*)	This study
JMN132	LY, Δ*R95J1_00450* (*pinZ*)	This study
JMN135	LY, Δ*R9J51_00370* (*pinV*)	This study
JMN136	LY, Δ*R9J51_00375* (*pinW*)	This study
JMN139	LY, Δ*R9J51_00455* (*pinU*)	This study
JMN140	LY, Δ*R9J51_02980* (*ferA*)	This study
Plasmids		
pAK405	Allele exchange plasmid for sphingomonads, Kan^R, *rpsL* (Sm^S)	(11)
pKMW3	Barcoded *mariner* transposon delivery vector	(10)
pJM480	pAK405, Δ*R9J51_00455* (*pinX*)	This study
pJM481	pAK405, Δ*R9J51_00470* (*ligC*)	This study
pJM482	pAK405, Δ*R9J51_15355* (*pinY*)	This study
pJM483	pAK405, Δ*R95J1_00450* (*pinZ*)	This study
pJM484	pAK405, Δ*R9J51_00370* (*pinV*)	This study
pJM485	pAK405, Δ*R9J51_00375* (*pinW*)	This study
pJM486	pAK405, Δ*R9J51_00455* (*pinU*)	This study
pJM487	pAK405, Δ*R9J51_02980* (*ferA*)	This study
pJM491	pET-24a(+), NdeI-XhoI fragment containing *pinU*	This study
pJM492	pET-28a(+), NdeI-XhoI fragment containing *pinZ*	This study

Additional References:

1. Ralph J, Helm RF, Quideau S. 1992. Lignin–feruloyl ester cross-links in grasses. Part 2. Model compound syntheses. J Chem Soc Perkin 1 2971–2980.

2. Eklund PC, Riska AI, Sjöholm RE. 2002. Synthesis of *R*-(–)-imperanene from the natural lignan hydroxymatairesinol. J Org Chem 67:7544–7546.

3. He Y, Jia Y, Lu F. 2020. New products generated from the transformations of ferulic acid dilactone. Biomolecules 10.

4. Quideau S, Ralph J. 1993. Synthesis of 4,8-bis(4-hydroxy-3-methoxyphenyl)-3,7-dioxabicyclo[3.3.0]octan-2-ones and determination of their relative configuration via long-range proton couplings. J Chem Soc Perkin 1 653–659.

5. Kuatsjah E, Zahn M, Chen X, Kato R, Hinchen DJ, Konev MO, Katahira R, Orr C, Wagner A, Zou Y, Haugen SJ, Ramirez KJ, Michener JK, Pickford AR, Kamimura N, Masai E, Houk KN, McGeehan JE, Beckham GT. 2023. Biochemical and structural characterization of a sphingomonad diarylpropane lyase for cofactorless deformylation. Proc Natl Acad Sci U S A 120:e2212246120.

6. Fukuhara Y, Kamimura N, Nakajima M, Hishiyama S, Hara H, Kasai D, Tsuji Y, Narita-Yamada S, Nakamura S, Katano Y, Fujita N, Katayama Y, Fukuda M, Kajita S, Masai E. 2013. Discovery of pinoresinol reductase genes in sphingomonads. Enzyme Microb Technol 52:38–43.

7. Nakatsubo T, Mizutani M, Suzuki S, Hattori T, Umezawa T. 2008. Characterization of *Arabidopsis thaliana* pinoresinol reductase, a new type of enzyme involved in lignan

biosynthesis. J Biol Chem 283:15550–15557.

8. Hwang JK, Moinuddin SGA, Davin LB, Lewis NG. 2020. Pinoresinol-lariciresinol reductase: Substrate versatility, enantiospecificity, and kinetic properties. Chirality 32:770–789.

9. Dinkova-Kostova AT, Gang DR, Davin LB, Bedgar DL, Chu A, Lewis NG. 1996. (+)-Pinoresinol/(+)-lariciresinol reductase from *Forsythia intermedia*. Protein purification, cDNA cloning, heterologous expression and comparison to isoflavone reductase. J Biol Chem 271:29473–29482.

10. Wetmore KM, Price MN, Waters RJ, Lamson JS, He J, Hoover CA, Blow MJ, Bristow J, Butland G, Arkin AP, Deutschbauer A. 2015. Rapid quantification of mutant fitness in diverse bacteria by sequencing randomly bar-coded transposons. MBio 6:e00306–15.

11. Kaczmarczyk A, Vorholt JA, Francez-Charlot A. 2012. Markerless gene deletion system for sphingomonads. Appl Environ Microbiol 78:3774–3777.
